# Sex and gender differences in memory in epilepsy: a systematic review and meta-analysis

**DOI:** 10.1186/s13293-025-00797-2

**Published:** 2025-12-13

**Authors:** Paula Tormos-Pons, Esperanza González-Bono, Irene Cano-López

**Affiliations:** Institut d’Investigacio en Psicologia dels Recursos Humans, del Desenvolupament Organitzacional i de la Qualitat de Vida Laboral (IDOCAL)/Department of Psychobiology, Psychology Center, Universitat de València, Valencia, Spain

**Keywords:** Epilepsy, Sex differences, Gender differences, Memory, Meta-analysis

## Abstract

**Background:**

Memory impairments are highly prevalent in patients with epilepsy, yet important gaps remain in the understanding of potential sex and gender differences. This systematic review and meta-analysis aim to synthesize the available evidence on sex and gender differences in memory functioning in adults and children with epilepsy, and to explore the relevance of the epilepsy type, the side of seizure focus, the hemispheric dominance for language, the educational level and the age group in these differences.

**Methods:**

The study followed PRISMA guidelines and was registered in PROSPERO (CRD420251006928). Studies were retrieved from Web of Science, PubMed/MEDLINE, Embase, and Scopus.

**Results:**

The systematic search yielded 1,261 records, from which 32 studies were selected. Women scored higher than men in immediate verbal memory, both at baseline (*g* = 0.34; 95% CI = 0.23, 0.44; *p* < 0.0001) and after epilepsy surgery (*g* = 0.30; 95% CI = 0.15, 0.44; *p* < 0.0001). This advantage was also observed in delayed verbal memory, at baseline (*g* = 0.30; 95% CI = 0.19, 0.41; *p* < 0.0001) and after surgery (*g* = 0.25; 95% CI = 0.09, 0.41; *p* = 0.0018). In contrast, men outperformed women in immediate visual memory, both before (*g* = -0.13; 95% CI = -0.22, -0.03; *p* = 0.01) and after surgery (*g* = -0.17; 95% CI = -0.33, -0.01; *p* = 0.04). No significant differences were observed in working memory or delayed visual memory. Effect sizes favoring women in verbal memory were significantly smaller in studies including only patients with temporal lobe epilepsy compared to mixed epilepsy types. The effect size for postsurgical delayed verbal memory was moderated by the side of seizure focus: studies including a greater proportion of patients with left-hemisphere epilepsy showed poorer postsurgical delayed verbal memory. Hemispheric dominance for language, age, and educational level did not moderate sex-gender differences in memory.

**Conclusions:**

These findings underscore the importance of incorporating sex and gender variables in neuropsychological assessment and intervention planning, offering evidence-based recommendations.

## Highlights

Women had better immediate and delayed verbal memory than men before and after surgery.

Men had better immediate visual memory than women before and after surgery.

Women and men did not differ in working memory or delayed visual memory.

Studies including a greater proportion of patients with left-hemisphere epilepsy showed poorer postsurgical delayed verbal memory.

Age did not moderate gender differences, but pediatric samples were limited.

## Background

Epilepsy is a neurological disorder affecting approximately 50 million individuals worldwide [1]. Beyond the predisposition to recurrent seizures, epilepsy is associated with a wide range of neurobiological, cognitive, psychological, and social consequences [2]. Its incidence shows sex-specific patterns, with a higher overall vulnerability in men [3], despite the higher prevalence of more complex and drug-resistant seizure types in women [4]. Increasing evidence suggests that sex and gender play a significant role in shaping these outcomes, including clinical presentation and the effects of antiseizure medications across the lifespan [5–8]. Among the most frequent and impactful comorbidities are cognitive impairments, with memory being one of the most affected domains in both adults and children [9, 10], especially in patients with temporal lobe epilepsy (TLE), in which seizures are triggered in brain networks particularly relevant for memory consolidation [11].

Despite the high prevalence of memory impairments in this clinical population, there remain gaps in the understanding of potential sex- and gender-related differences. Biological factors such as variations in sex hormone levels may shape distinct patterns of memory functioning in women and men [12]. Several studies indicate that sex hormones play a fundamental role in sex differences in brain morphology as well as in structural and functional connectivity, which may contribute to differential vulnerability to various pathological processes [13–16]. Furthermore, sex hormones have also been implicated in sex differences in seizure susceptibility: progesterone is an anticonvulsant hormone, estrogen is considered both proconvulsant and anticonvulsant depending on the physiological status, and testosterone produces proconvulsant and anticonvulsant effects depending on the seizure type [17]. These effects may be modulated, at least in part, through the interactions between sex hormones and the hypothalamic-pituitary-adrenal (HPA) axis [4, 8, 18, 19]. In fact, epilepsy has been related to a dysregulation of both the hypothalamic-pituitary-gonadal (HPG) axis and the HPA axis [20–22]. Structures of the limbic system, such as the hippocampus - which play a central role in memory processes- exhibit high densities of receptors for estrogens, progesterone, testosterone, and cortisol, making them especially sensitive to hormonal fluctuations [23–25]. The actions of sex and stress hormones, particularly in modulating synaptic organization within the hippocampus and prefrontal cortex, may have important implications for memory function [26, 27].

Psychological and social factors may also contribute to these differences. For example, depression and anxiety are highly prevalent in patients with epilepsy [28], with women showing greater vulnerability to these disorders than men [29, 30]. In this regard, several studies have demonstrated that anxiety and depression are linked to poorer memory performance [31, 32]. Cognitive reserve may also contribute to better memory functioning by mitigating the impact of functional brain connectivity alterations [33, 34]. Gender differences have been described in access to cognitively enriching opportunities, with women often facing reduced access due to traditional gender roles that limit educational attainment, professional development, and participation in intellectually stimulating activities [35]. In addition, it has been suggested that women and men may benefit from cognitive reserve through different patterns of functional connectivity or specific compensatory strategies [36]. Taken together, biological and psychosocial influences may interact with sex and gender, shaping differentiated profiles of memory vulnerability or resilience.

Despite the growing recognition of sex- and gender-related variability in epilepsy, no prior meta-analysis has evaluated its influence on memory functioning. Consequently, the present study aims to examine sex and gender differences in memory in patients with epilepsy, by carrying out a systematic review and meta-analysis of the literature and exploring the relevance of clinical variables such as the epilepsy type, the side of seizure focus and the hemispheric dominance for language, as well as demographic variables such as the age group and the educational level in these differences. Understanding how sex- and gender-related factors modulate disease mechanisms -particularly cognitive outcomes- can provide valuable insights for clinical practice. Considering these differences may enhance the accuracy of neuropsychological assessments and presurgical predictions by refining the characterization of cognitive profiles and outcome patterns within each sex and gender group, guide the development of more individualized rehabilitation and surgical strategies [37], and foster sex- and gender-informed approaches to clinical decision-making, ultimately contributing to improved quality of life in patients with epilepsy.

## Methods

### Search strategy and information sources

The systematic review was conducted according to the Preferred Reporting Items for Systematic Reviews and Meta-Analyses (PRISMA) recommendations [38], and it was registered in PROSPERO (CRD420251006928).

The search was conducted in the following databases: Web of Science, Pubmed/MEDLINE, Embase, and Scopus, from inception to April 16, 2024. The terms used were formulated as follows: (epilep* OR seizure OR convulsion) AND (sex* difference* OR gender OR testosterone OR androgen OR estradiol OR estrogen OR progesterone) AND (memory). These keywords were examined in the title section, keywords, and abstract. In addition, a reverse search was performed by reviewing the reference lists of systematic reviews and empirical studies to identify records not indexed in these databases. The search was not limited by date or age.

### Eligibility criteria

The inclusion criteria were as follows: (a) original and empirical studies published in peer-reviewed journals; (b) studies focused on sex or gender differences on objective measures of memory in people with epilepsy; (c) studies including children and adults with a diagnosis of epilepsy; (d) studies with data from patients with epilepsy separated from other groups of patients included in the study; and (e) studies written in English or Spanish. We excluded: (a) animal studies; (b) studies with mixed data on people with epilepsy and other groups of patients; (c) studies exclusively including patients with psychogenic non-epileptic seizures (PNES); (d) studies that have only subjective memory measures; (e) books and book chapters, single-case studies, reviews, meta-analyses, study protocols, editorials, or conference abstracts. Studies were not excluded due to other comorbid conditions, the etiology of epilepsy, or epilepsy treatments.

### Study selection

Duplicate references were removed using EndNote X9 and Rayyan software [39]. Two authors (PTP and ICL) independently assessed titles and abstracts for possible agreement with the inclusion criteria. Discrepancies were resolved through critical discussion among the three authors (PTP, ICL, and EGB) until 100% agreement was reached. Next, each researcher individually assessed the full text of the articles selected to determine whether they met the inclusion criteria. Studies that failed to meet the inclusion criteria were excluded.

### Data extraction

Two authors (PTP and ICL) independently extracted data from all the revised studies as follows: (a) reference; (b) study design; (c) sample size and distribution by sex/gender; (d) age; (e) epilepsy type and age at onset; (f) seizure frequency; (g) treatment; (h) memory measures; and (i) results about sex/gender differences in memory.

### Data synthesis

Data extracted from the reviewed studies were divided into three categories: (a) characteristics of the sample (i.e., age group, epilepsy type and treatment); (b) memory assessment; and (c) sex/gender differences in memory (i.e., working memory, immediate verbal memory, delayed verbal memory, immediate visual memory, and delayed visual memory).

### Methodological quality assessment

Two reviewers (ICL and PTP) independently assessed the methodological quality of the studies, with any discrepancies discussed in collaboration with a third author (EGB). The Mixed Methods Appraisal Tool (MMAT) [40] was used for this evaluation, as it is specifically designed for the critical appraisal of systematic reviews that include various study designs. For quantitative non-randomized studies, the MMAT assesses five key issues: the representativeness of the sample, ensuring that participants adequately reflect the target population; the appropriateness of the measurements, evaluating the validity and reliability of the instruments used; the completeness of outcome data, ensuring that data loss does not compromise the validity of the findings; the consideration of confounding variables, determining whether the study properly accounts for factors that may influence the results; and the adequacy of intervention administration or exposure occurrence, ensuring consistency and accuracy in the implementation of the treatment or exposure within the study population. Regarding the completeness of outcome data, we considered outcome data to be acceptably complete when at least 80% of the data were available.

### Meta-analyses

Meta-analyses were conducted using the metafor package [41] within the R software environment [42]. The objective was to examine memory differences between women and men. The cognitive domains analyzed included working memory, immediate and delayed verbal memory, and immediate and delayed visual memory. A hierarchy of assessment instruments was established for each domain, prioritizing the most frequently used measures across the included studies. When multiple measures were reported within a single study, the highest-ranked instrument was selected for data extraction. In studies assessing the effects of epilepsy surgery on memory, both baseline (pre-surgical) differences and post-surgical outcomes were examined in separate meta-analyses. In these cases, the first available postsurgical assessment was employed, corresponding to six or twelve months after surgery, depending on the study. If mean and standard deviation (SD) values were unavailable, corresponding authors were contacted to request the data, following best practices in meta-analyses [43]. Studies for which data could not be retrieved were excluded from the analysis.

The meta-analysis employed a random-effects model for continuous data, using inverse variance with a 95% confidence interval. This approach accounts for potential variability across studies due to differences in sample characteristics, cognitive assessment tools, and methodological approaches [44]. Effect sizes were calculated using Hedges’ g (adjusted standardized mean difference [SMD]), with a small effect size defined as 0.20 or lower (range 0–0.20); moderate = 0.50 (range 0.30–0.70); and large = 0.80 (≥ 0.80) [45, 46]. Potential outliers were identified using the Galbraith plot method [47]. To assess potential publication bias, Egger’s test for funnel plot asymmetry [48] was applied in meta-analyses including 10 or more studies, as recommended [49]. In cases where asymmetry was detected, the Trim and Fill method was employed to estimate the number of potentially missing studies and to adjust the overall effect size accordingly [50]. Meta-regression analyses were conducted based on epilepsy type (TLE or mixed/other epilepsy types), side of seizure focus (proportion of patients with left-hemisphere seizure focus, with the remaining proportion representing right-hemisphere cases), hemispheric language dominance (proportion of patients with typical dominance), age group (children or adults), and educational level (years of education).

## Results

### Study selection

**Fig. 1:**
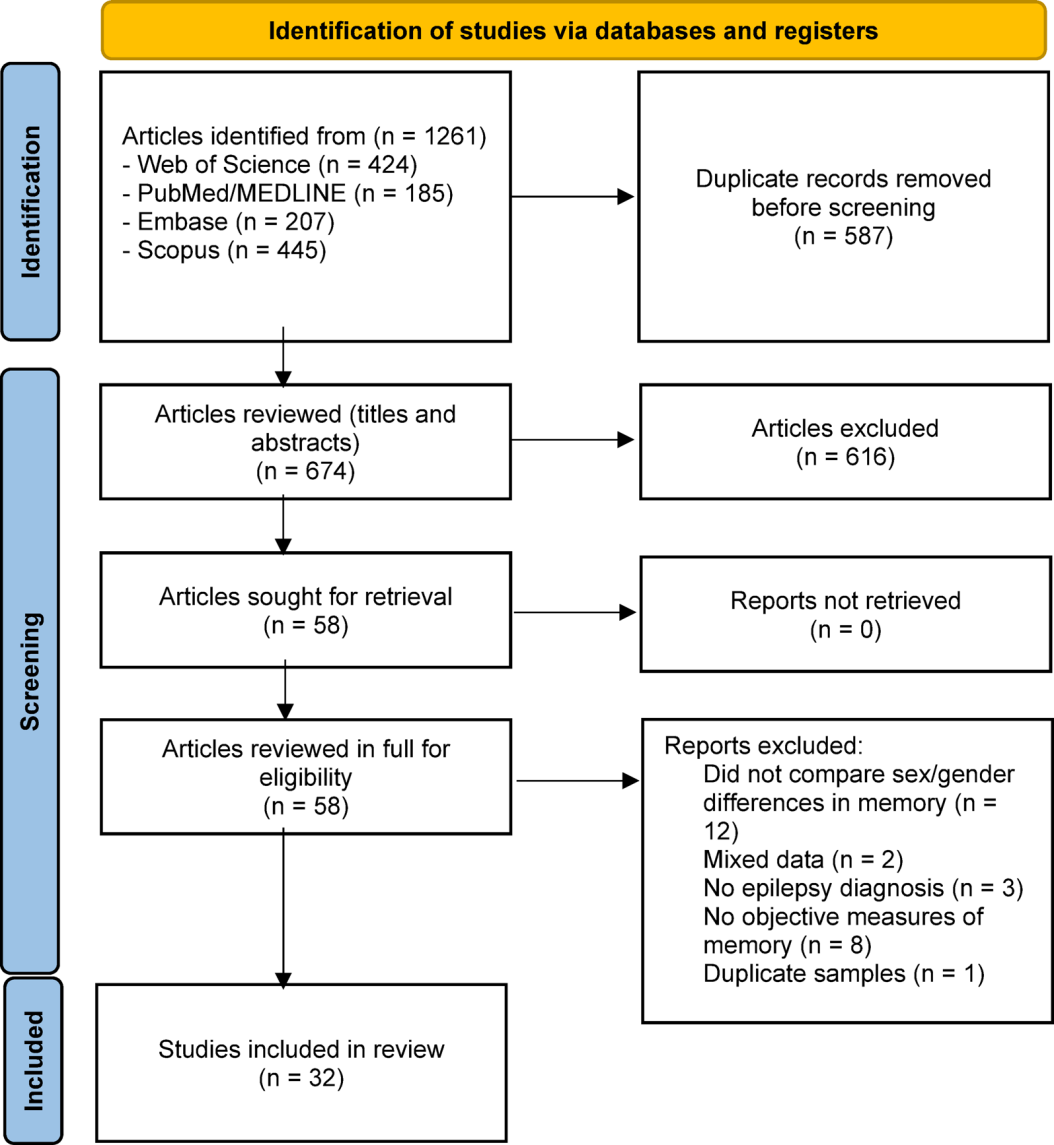
Flow diagram of the identification and selection of studies

The initial search yielded a total of 1261 articles – of which 587 were identified as duplicates. After reviewing the titles, abstracts, and keywords of the remaining 674 articles, a further 616 articles were excluded. Of the remaining 58 articles that were reviewed in full, 26 were excluded for six reasons: studies did not compare sex/gender differences in memory (*n* = 12); studies with mixed data on people with epilepsy and other groups of neurological patients that did not report separate data for patients with epilepsy (*n* = 2); studies with a sample without epilepsy diagnosis (*n* = 3); studies without objective memory measures (*n* = 8), and studies with duplicate samples (*n* = 1). Regarding duplicate samples, the study by Operto et al. [51] used the same sample as the article by Operto et al. [52]. According to the Cochrane Handbook for Systematic Reviews of Interventions, duplicate publications can introduce substantial bias into meta-analyses [53]. Since only the article by Operto et al. [52] provided sufficient data for meta-analytic inclusion, whereas Operto et al. [51] lacked the necessary information, the former was selected for analysis. Thus, 32 articles [52, 54–84] were included in the review. The studies included are marked with the symbol (*) in the references section. Figure 1 shows the flow diagram of the identification and selection of studies. Table 1 includes a summary of the selected studies. In the reviewed studies, participants were labeled as ‘women’ and ‘men’ based on phenotypic sex. Consequently, we refer to ‘sex’ when describing study samples but adopt ‘sex/gender’ in our interpretations, acknowledging that (1) biological and social influences are often intertwined and (2) current evidence does not allow us to disentangle their specific impacts.

**Table 1:** Summary of the included studies

Study	Study design	Sample size; sex/gender	Age group (mean age ± SD)	Epilepsy type and age at onset	Seizure frequency	Treatment	Memory measures	Results
Baxendale et al. [54]	Cross-sectional	*N* = 382 patients with epilepsy Women: *n* = 213 (55.71%) Men: *n* = 169 (44.24%)	Adults; total mean = 32.90 ± 9.20 years Women: 32.90 ± 9.60 years Men: 32.70 ± 8.60 years	Drug-resistant TLE with hippocampal sclerosis Total mean age onset = 8.10 ± 4.90 years; women: 7.70 ± 5.00 years; men: 7.60 ± 4.90 years	Total mean: n.s. All participants: persistent seizures Women: n.s. Men: n.s.	Total mean ASMs: n.s.; the majority of patients were taking ≥ 2 ASMs; women: n.s.; men: n.s. Surgery: no	AMIPB: immediate and delayed verbal and visual memory 1 assessment: basal (T0)	*Women vs. men*: - N.s.d.
Baxendale et al. [55]	Longitudinal	*N* = 70 patients with epilepsy (data available for *n* = 68): Women: 42 (61.76%) Men: *n* = 26 (38.23%)	Adults; total mean: 33.84 ± 9.01 years Women: n.s. Men: n.s.	Drug-resistant TLE Total mean age onset = 12.24 ± 9.29 years; women = n.s.; men = n.s.	Total mean: n.s. Women: n.s. Men: n.s.	Total mean ASMs: n.s.; T0: all patients were on multiple ASMs; T3: patients not seizure-free were taking ≥ 2 ASMs; among seizure-free patients, 5 were off medication, 19 were taking 1 ASM, and 6 continued with ≥ 2 ASMs; women: n.s.; men: n.s. Surgery: ATL	AMIPB: immediate verbal and visual memory 4 assessments: basal (T0) and 3 months (T1), 1 year (T2), and 5 years (T3) after surgery	*Women vs. men*: - T0: n.s.d. - Change from T0 to T2 and T3: Patients with progressive memory decline were more likely to be female and to experience seizures after surgery
Baxendale [56]	Longitudinal	*N* = 372 patients with epilepsy Women: *n* = 202 (54.30%) Men: *n* = 170 (45.70%)	Adults; total mean = 33.44 years; SD: n.s. Women = 32.80 years; SD: n.s. Men = 34.20 years; SD: n.s.	TLE with hippocampal sclerosis Total mean age onset = 10.15 ± 8.59 years; women = 9.10 ± 7.20 years; men = 11.40 ± 10.00 years	Total mean: n.s. Women: n.s. Men: n.s.	Total mean ASMs: n.s. All patients were taking at least 1 ASM; women: n.s.; men: n.s. Surgery: standardized right or left temporal lobe resection	AMIPB/BMIPB: immediate and delayed verbal memory, and immediate visual memory 2 assessments: basal (T0) and 1 year after surgery (T1)	*Women vs. men*: - T0: >verbal memory (immediate story recall, delayed story recall, and verbal recall following distraction). - T0: < visual memory (visual recall following distraction). - From T0 to T1: ↑ postsurgical verbal memory decline (immediate and delayed story recall). N.s.d. in the rest of variables
Bengner et al. [57]	Cross-sectional	*N* = 69 patients with epilepsy Women: *n* = 40 (57.97%) Men: *n* = 29 (42.03%)	Adults; total mean = 37.09 ± 13.26 years Women = n.s. Men = n.s.	TLE (*n* = 49) and generalized epilepsy (*n* = 20) Epilepsy duration: women: median = 16 years, quartiles = 7, 27; men: median = 15 years, quartiles: 8, 21	Total mean = n.s. Women = n.s. Men = n.s.	Total mean ASMs: 1.6 ± 0.76; women: 1.6 ± 0.8; men: 1.6 ± 0.7 Surgery: no	Visual memory tasks designed ad hoc (face recognition): immediate and delayed visual recognition 2 assessments: day 1 (T0) and 24 h after (T1) to assess delayed recognition	*Women vs. men*: - From T0 to T1: ↑ delayed face recognition
Bengston et al. [58]	Longitudinal	*N* = 70 patients with epilepsy Women: *n* = 38 (54.29%) Men: *n* = 32 (45.71%)	Adults; total mean = 33.74 ± 9.21 years Women = 34.03 ± 9.55 years Men = 33.40 ± 8.78 years	Drug-resistant TLE Total mean age onset: 13.12 ± 9.87 years; women: 12.41 ± 8.75 years; men: 13.97 ± 11.00 years	Total mean: n.s. Women: n.s. Men: n.s.	Total mean ASMs: n.s.; women: n.s.; men: n.s. Surgery: en bloc left or right ATL	WMS-III, LM subtest: delayed verbal memory 2 assessments: basal (T0) and 6 months after surgery (T1)	*Women vs. men*: - T0: n.s.d. - From T0 to T1: ↑ postoperative verbal memory performance *Women*: - N.s.r. between hippocampal volume and memory *Men*: - In left TLE patients: right hippocampal volume was associated with better verbal memory performance - In right TLE patients: n.s.r. between hippocampal volume and memory
Berenbaum et al. [59]	Longitudinal	*N* = 57 patients with epilepsy Women: *n* = 30 (52.63%) Men: *n* = 27 (47.37%)	Adults; total mean = 31.16 ± 8.69 years Women = 30.70 ± 8.91 years Men = 31.67 ± 8.41 years	Drug-resistant left TLE Total mean age onset = 13.13 ± 11.54 years; women = 13.02 ± 11.69 years; men = 13.26 ± 11.38 years	Total mean: n.s. Women: n.s. Men: n.s.	Total mean ASMs: n.s.; women: n.s.; men: n.s. Surgery: left ATL	CVLT: immediate and short-term verbal memory WAIS-R, Digit Span Subscale: working memory 2 assessments: basal (T0) and 6 months after surgery (T1)	*Women vs. men*: - T0 and T1: >immediate and short-term verbal memory (regardless of the status of the resected hippocampus), and more likely to use semantic clustering encoding strategy - From T0 to T1: n.s.d. in postsurgical memory changes - N.s.d. in working memory
Berger et al. [60]	Longitudinal	*N* = 177 patients with epilepsy Women: *n* = 95 (53.67%) Men: *n* = 82 (46.33%)	Adults; total mean = 37.45 ± 12.05 years Women = 37.76 ± 11.00 years Men = 37.10 ± 13.16 years	TLE Total mean age onset = 16.98 ± 11.61 years; women = 17.18 ± 11.57 years; men = 16.75 ± 11.65 years	Total mean: n.s. Women: n.s. Men: n.s.	Total mean ASMs: n.s.; women: n.s.; men: n.s. Surgery: left and right ATL, including SAH, LES, amygdalectomy sparing hippocampus, and Hippocampectomy sparing amygdala	VLMT, adapted from RAVLT: verbal learning and delayed verbal memory NVLT: Nonverbal learning and delayed visual recognition 2 assessments: basal (T0) and 1 year after surgery (T1)	*Women vs. men*: - T0 and T1: >verbal learning and delayed verbal memory, independently of the side of surgery. These differences remained significant in the subsample of patients with HS who were seizure-free after surgery - T0 and T1: N.s.d. in nonverbal learning and delayed nonverbal memory
Berger et al. [61]	Cross-sectional	*N* = 233 patients with epilepsy Women: *n* = 116 (49.79%) Men: *n* = 117 (50.21%)	Adults; total mean: 35.53 ± 12.22 years Women = 36.09 ± 11.35 years Men = 34.97 ± 13.07 years	FLE and TLE Total mean age onset = 17.41 ± 11.76 years; women = 17.34 ± 11.06 years; men = 17.47 ± 12.45 years	Total mean: n.s. Women: n.s. Men: n.s.	Mean ASMs = 1.79 ± 0.66; women = 1.67 ± 0.62; men = 1.92 ± 0.70 Surgery: no	VLMT, adapted from RAVLT: verbal learning and delayed verbal memory WMS-III, Digit Span Subscale: working memory 1 assessment: basal (T0)	*Women vs. men*: - Total sample: >verbal learning and delayed verbal memory - TLE group: >delayed verbal memory - FLE group: n.s.d. in delayed verbal memory - TLE and FLE groups: n.s.d. in working memory
Bjørnæs et al. [62]	Longitudinal	*N* = 91 patients with epilepsy Women: *n* = 45 (49.45%) Men: *n* = 46 (50.55%)	Adults; total mean = 32.19 ± 9.57 years Women = 32.90 ± 9.87 years Men = 31.47 ± 9.26 years	TLE Total mean age onset = 12.89 ± 9.11 years; women = 12.13 ± 8.82 years; men = 13.67 ± 9.40 years	Total mean (FIAS): 13.99; SD: n.s. Women: 18.85; SD: n.s. Men: 9.02; SD: n.s.	Mean ASMs: n.s.; women: n.s.; men: n.s. Surgery: ATL; 1 patient SAH; 2 patients with minor basolateral right frontal lobe excisions	Tasks adapted from Luria test: verbal memory: learning, trials to criterion, short and long verbal delay, percentage retention Tasks adapted from Jones-Gotman: Visual memory: learning, best results during acquisition, short and long visual learning delay, percentage retention TPT: number of blocks and spatial localization recalled without delay BVRT-R: visual working memory WAIS, Digit Span: working memory 3 assessments: basal (T0), 6 months (T1) and 2 years after surgery (T2)	*Women vs. men*: - T0, T1 and T2: >verbal learning in first trial (working memory) - T0, T1 and T2: need fewer number of verbal learning trials to criterion (speed of acquisition) - T0 and T1: < long-delay visual learning - N.s.d. in other indices *Women*: - From T0 to T1 and from T0 to T2: no significant changes in long-delay verbal memory - Subgroup of left TLE: from T0 to T1:↑in long-delay visual learning - Subgroup of left TLE: from T0 to T2:↑in visual learning best result and long-delay visual learning *Men*: - From T0 to T1 and from T0 to T2: ↓ in long-delay verbal memory - Subgroup of left TLE: no significant changes in visual learning *Women with left TLE vs. women with right TLE*: - T0: < long-delay verbal memory *Men with left TLE vs. men with right TLE and women*: - T1 and T2: < long-delay verbal memory
Cánovas et al. [63]	Cross-sectional	*N* = 25 patients with epilepsy Women: *n* = 14 (56.00%) Men: *n* = 11 (44.00%)	Adults; total mean = 42.12 ± 10.23 years Women = n.s. Men = n.s.	Drug-resistant mesial TLE Total mean age onset = 17.04 ± 11.65 years; women: = n.s; men = n.s.	Total mean: n.s. Women: n.s. Men: n.s.	Total mean ASMs: n.s.; women: n.s.; men: n.s. Type of ASMs: clobazam (56%), oxcabazepine (48%), levetiracetam (36%), carbamazepine (32%), lamotrigine (24%), phenobarbital (20%), topiramate (12%), valproic acid (12%), others (8%), others 8%; women: n.s.; men: n.s. Surgery: no	“Boxes Room” a virtual reality version of the holeboard: Spatial navigation and visual learning and memory 1 assessment: basal (T0)	*Women vs. men*: - N.s.d.
Chai et al. [64]	Cross-sectional	*N* = 90 patients with epilepsy Women: *n* = 47 (52.20%) Men: *n* = 43 (47.80%)	Adults; total mean = 61.00 ± 10.52 years Women = n.s. Men = n.s.	Epilepsy type: n.s. Age onset: early Percentage of age onset: early onset (< 60 years): n = = 73 (81.10%); late onset (≥ 60 years): n = = 17 (18.90%); women = n.s.; men = n.s.	Total mean: 3.00 ± 17.78 years Women: n.s. Men: n.s.	Number of ASMs: ASMs and percentage of patients taking it:, 0 (6.70%), 1 (38.90%), 2 or more (54.40%); women: n.s.; men: n.s. Surgery: no	AVLT, adapted from CVLT: immediate verbal recall The Stick Test adapted from the Stick Design Test: nonverbal memory (specifically the recall score) 1 assessment: basal (T0)	*Women vs. men*: - N.s.d.
Davies et al. [65]	Longitudinal	*N* = 203 patients with epilepsy Women: *n* = 110 (54.19%) Men: *n* = 93 (45.81%)	Adults; total mean = 31.33 ± 9.37 years Women: 32.23 ± 9.18 years Men: 30.26 ± 9.59 years	Drug-resistant TLE Total mean age onset = 11.92 ± 11.01 years; women: 12.27 ± 11.45 years; men: 11.49 ± 10.49 years	Total mean: n.s. Women: n.s. Men: n.s.	Total mean ASMs: n.s.; women: n.s.; men: n.s. Surgery: ATL	CVLT: verbal learning, short-term verbal memory, delayed verbal memory and percentage loss 2 assessments: basal (T0) and 6 months after surgery (T1)	*Women vs. men*: - N.s.d. in presurgical memory or postsurgical statistical changes *Multiple regression analyses*: - RCIs: the risk of verbal learning decline increased with a higher presurgical score, higher chronological age, lower IQ, left-sided resections and male sex
Grammaldo et al. [66]	Longitudinal	*N* = 82 patients with epilepsy Women: *n* = 39 (48.00%) Men: *n* = 43 (52.00%)	Adults; total mean = 34.1 ± 10.3 years Women = n.s. Men = n.s.	Drug-resistant TLE Epilepsy duration: 19.00 ± 11.80 years; women: n.s.; men: n.s.	Total mean: n.s. Women: n.s. Men: n.s.	Total mean ASMs: n.s.; in most cases, ≥ 2 ASMs; women: n.s.; men: n.s. Surgery: ATL	RAVLT: verbal learning, immediate and delayed verbal memory SRT: immediate verbal memory and delayed verbal memory recall ROCF: figural copy, immediate and delayed visual recall 3 assessments: basal (T0), 1 year (T1) and 2 years after surgery (T2)	*Multiple regression analyses: Women vs. men*: - From T0 to T2: Being a man was associated with greater improvement in delayed verbal memory recall (SRT), together with higher educational level - From T0 to T2: n.s.d. in RAVLT - From T0 to T2: Being a man was associated with greater improvement in immediate and visual recall, together with a younger age
Helmstaedter et al. [67]	Cross-sectional	*N* = 85 patients with epilepsy Women: *n* = 43 (50.59%) Men: *n* = 42 (49.41%)	Adults; total mean = 27.48 ± 9.49 years Women = 26.00 ± 9.00 years Men = 29.00 ± 10.00 years	Drug-resistant left TLE Total mean age onset = 10.51 ± 7.00 years; women = 11.00 ± 7.00 years; men = 10.00 ± 7.00 years	Total mean: n.s. Women: n.s. Men: n.s.	Total mean ASMs: n.s.; women: n.s.; men: n.s. Surgery: no	VLMT, adapted from RAVLT: verbal learning, delayed free recall and delayed recognition DCS-R: immediate visual memory BVRT: short-term visual retention 1 assessment: basal (T0)	*Women vs. men*: - >verbal memory: verbal learning and delayed free recall, particularly in those patients with atypical language representation - < visual memory: visual learning (DCS-R) and short-term visual retention (BVRT), especially in those patients with left language representation
Helmstaedter et al. [68]	Longitudinal	*N* = 169 patients with epilepsy Women: *n* = 75 (44.38%) Men: *n* = 94 (57.40%)	Adults; total mean = 28.78 ± 10.50 years Women = 27.80 ± 10.50 years Men = 30.00 ± 10.50 years	Left TLE Total mean age onset = 11.45 ± 8.50 years; women = 10.50 ± 8.00 years; men = 12.20 ± 8.90 years	Total mean: n.s. Women: n.s. Men: n.s.	Total mean ASMs: n.s.; women: n.s.; men: n.s. Surgery: SAH, LES or ATL of the left hemisphere	VLMT, adapted from RAVLT: A total verbal memory score using verbal learning and delayed verbal memory DCS-R: immediate visual memory 2 assessments: basal (T0) and 1 year after surgery (T1)	*Women vs. men*: - T0: >verbal memory, mainly to the subgroup with incomplete left hemisphere or bilateral language representation and also with a trend in the right language representation subgroup - T0: n.s.d. in visual learning - From T0 to T1: n.s.d. in postsurgical verbal memory changes - From T0 to T1: ↑ postoperative visual learning after surgery than men who showed greater impairment, especially when they were not left dominant
Hernández et al. [69]	Cross-sectional	*N* = 32 patients with epilepsy Girls: *n* = 12 (37.50%) Boys: *n* = 20 (62.50%)	Children; total mean = n.s.; range: 7–16 years Girls = 11.64 ± 2.88 years Boys = n.s.; range: 7.67–15.59 years	TLE, FLE and generalized epilepsy Total mean age onset = n.s.; girls = 8.35 ± 3.42 years; boys = n.s.; range = 3.16–14.24 years	Total mean: n.s.; occasional seizures (53.13%) and no seizures for at least 1 year (46.88%) Girls: n.s. Boys: n.s.	Total mean ASMs: n.s.; distribution of ASMs: 0 ASMs (6.25%), 1 ASM (53.13%), more than 1 ASM (40.63%); girls: n.s.; boys: n.s. Surgery: no	SOPT: working memory 1 assessment: basal (T0)	*Girls vs. boys*: - N.s.d. in working memory
Höller et al. [70]	Longitudinal	*N* = 97 Women: n.s. Men: n = n.s.	Adults; total mean = 27.50 years; SD: n.s. Women = n.s. Men = n.s.	Focal epilepsy (64.21%), generalized epilepsy (13.68%), no epilepsy (12.63%) and unclear diagnosis (9.47%) Total mean age onset: median (range) = 19.00 (0–62 years); women = n.s.; men = n.s.	Total mean: n.s. Women: n.s. Men: n.s.	Mean ASMs = n.s.; distribution of ASMs: 0 ASMs (22.68%), 1 ASM (42.27%), 2 ASMs (22.68%), 3 ASMs (11.34%); women = n.s.; men = n.s. Surgery: no	Virtual Reality Task: immediate and delayed episodic memory examined on subscales of remembered semantic contents (what, details), information in time (when), and space (where), spatial allocentric and egocentric memory 7 assessments: twice a day in 4 consecutive days (only first evaluation was once a day)	*Women vs. men*: - N.s.d. in immediate and delayed memory
Hudry et al. [71]	Cross-sectional	*N* = 38 patients with epilepsy Women: *n* = 20 (52.63%) Men: *n* = 18 (47.37%)	Adults; total mean = 32.80 ± 8.80 years Women = 33.76 ± 8.76 Men = 32.05 ± 7.7	Drug-resistant TLE Total epilepsy duration = 23.20 ± 8.30; women = 23.79 ± 8.72 years; men = 22.35 ± 7.55 years	Total mean: n.s. Women: n.s. Men: n.s.	Total mean ASMs: n.s.; women: n.s.; men: n.s. Surgery: no	Delayed Odour-matching Task: olfactory short-term recognition memory 1 assessment: basal (T0)	*Women vs. men*: - < false alarm rate and > discrimination ability, regardless of the side of the seizure focus
Ladavas et al. [72]	Cross-sectional	*N* = 75 patients with epilepsy Women: *n* = 30 (40.00%) Men: *n* = 45 (60.00%)	Adults; total mean = 30.55 years; SD = n.s. Women = n.s. Men = n.s.	TLE and FLE Total mean age onset = 16.70 years; SD = n.s.; women = n.s.; men = n.s.	Total mean: n.s. Women: n.s. Men: n.s.	Total mean ASMs: n.s.; women: n.s.; men: n.s. Surgery: no	WB, Digit Span Subscale: verbal working memory CBT: visuo-spatial working memory Digit Span + 1: verbal learning and long-term memory RAVLT: delayed verbal memory CBT + 1: visuo-spatial learning and long-term memory ROCF: delayed visual memory 1 assessment: basal (T0)	*Women vs. men (TLE group)*: - N.s.d. in short- and long-term visual memory - N.s.d. in short-term verbal memory - N.s.d. in recency judgment - In left TLE: >verbal long-term memory (RAVLT) - In right TLE: < verbal long-term memory (Digit Span + 1)
Matešić et al. [73]	Cross-sectional	*N* = 57 patients with epilepsy Women: *n* = 33 (57.89%) Men: *n* = 24 (42.11%)	Adults; total mean = 32.00 ± 13.40 years Women = n.s. Men = n.s.	TLE Total mean age onset = n.s.; women = n.s.; men = n.s.	Total mean: n.s. Women: n.s. Men: n.s.	Mean ASMs = n.s.; women = n.s.; men = n.s. Surgery: no	ROCF: immediate and delayed visual recall 1 assessment: basal (T0)	*Women vs. men*: - N.s.d. in immediate and delayed visual recall
Operto et al. [52]	Longitudinal	*N* = 207 patients with epilepsy Girls: *n* = 87 (42%) Boys: *n* = 120 (58%)	Children; total mean = 10.35 ± 2.39 years Girls = n.s. Boys = n.s.	TLE = 68 (61%) FLE = 29 (26%) OLE = 15 (13%). All patients: new diagnosis of epilepsy and well-controlled epilepsy Total mean age onset = 9.53 ± 2.41; girls = n.s.; boys = n.s.	Total mean: 12.64 ± 10.58 per month Girls: n.s. Boys: n.s.	Total mean ASMs = n.s. All patients were using monotherapy girls: n.s.; boys: n.s. Type of ASMs: Levetiracetam: *n* = 58 (girls: *n* = 28; boys: *n* = 30), carbamazepine: *n* = 44 (girls: *n* = 19; boys: *n* = 25), ethosuximide: *n* = 22 (girls: *n* = 10; boys = 12), oxcarbazepine *n* = 23 (girls: *n* = 10; boys: *n* = 13), valproic acid *n* = 60 (girls: 20; boys: 40) Surgery: no	ROCF: figural copy and immediate visual recall 2 assessments: basal (T0) and 12 months after monotherapy(T1)	*Girls vs. boys*: - N.s.d.
Salvato et al. [74]	Longitudinal	*N* = 151 patients with epilepsy Women: *n* = 63 (41.72%) Men: *n* = 88 (58.28%)	Adults; total mean = 33.95 ± 10.19 years Women = n.s. Men = n.s.	Drug-resistant TLE Total epilepsy duration = 21.11 ± 10.41 years; women = n.s.; men = n.s.	Total mean: 13.50 ± 15.26 Women: n.s. Men: n.s.	Total mean ASMs = n.s.; women = n.s.; men = n.s. ASMs withdrawal: 41% of patients Surgery: ATL	PAL: verbal learning and immediate verbal memory SS: immediate and delayed verbal memory 4 assessments: basal (T0), 6 months (T1), 2 years (T2) and 5 years after surgery (T3)	*Binary logistic regression analyses: Women vs. men*: - From T0 to T3: ↑ likely to exhibit memory decline (SS) - From T0 to T3: subgroup with left TLE for longer duration: ↑ at risk of memory decline (SS) - N.s.d. in PAL
Schwartz and Dennerll [75]	Cross-sectional	*N* = 140 patients with epilepsy Girls: *n* = 55 (39.29%) Boys: *n* = 85 (60.71%)	Children; total mean = 12.09 ± 2.08 years Girls = n.s. Boys = n.s.	TLE Total mean age onset = n.s.; girls = n.s.; boys = n.s.	Total mean: n.s. Girls: n.s. Boys: n.s.	Total mean ASMs = n.s.; girls = n.s.; boys = n.s. Surgery: no	WISC, Digit Span Subscale: working memory BG test, Recall Subtest: immediate visual memory 1 assessment: basal (T0)	*Girls vs. boys*: - N.s.d. in immediate visual memory - >working memory scores
Smith et al. [76]	Cross-sectional	*N* = 51 patients with epilepsy Girls: *n* = 25 (49.02%) Boys: *n* = 26 (50.98%)	Children; total mean = 13.18 ± 2.88 years Girls = 14.1 ± 2.7 years Boys = 12.3 ± 3.1 years	Drug-resistant epilepsy; TLE: *n* = 23; ETLE: *n* = 28 Total mean age onset = 6.18 ± 4.06 years; girls = 7.10 ± 4.10; boys = 5.30 ± 4.10	Total mean: n.s. Girls: n.s. Boys: n.s.	Total mean ASMs = 2.00 ± 0.79; girls = 2.2 ± 0.8; boys = 1.8 ± 0.8 Surgery: no	CAVLT: verbal learning and delayed verbal memory SRT: immediate and delayed verbal memory ROCF: delayed visuospatial memory Face recognition: visual recognition memory 1 assessment: basal (T0)	*Girls vs. boys*: - >performance in delayed recall of stories (SRT) and in the word list learning (CAVLT) - N.s.d. in the visual memory tasks
Strauss et al. [77]	Cross-sectional	*N* = 24 patients with epilepsy Women: *n* = 16 (66.67%) Men: *n* = 8 (33.33%)	Adults; total mean = 23.31 ± 6.28 years Women = 21.71 ± 7.39 years Men = 26.50 ± 3.54 years	Drug-resistant left TLE and FLE Total mean age onset = 1.93 ± 3.16 years; women = 1.82 ± 3.54 years; men = 2.15 ± 2.42 years	Total mean: n.s. Women: n.s. Men: n.s.	Total mean ASMs = n.s.; women = n.s.; men = n.s. Surgery: no	WMS, LM subtest: immediate and delayed verbal memory WMS, VR subtest: immediate and delayed visual memory 1 assessment: basal (T0)	*Women vs. men*: - Differences in verbal and visual memory: n.r. *Interaction effects between sex and speech lateralization*: - Women with atypical language lateralization vs. women with typical language lateralization: < immediate verbal memory, immediate and delayed visual memory - Men: n.s.d. depending on language lateralization in memory
Strauss et al. [78]	Cross-sectional	*N* = 121 patients with epilepsy Women: *n* = 67 (55.37%) Men: *n* = 54 (44.63%)	Adults; total mean = 29.25 ± 9.88 years Women = n.s. Men = n.s.	Drug-resistant epilepsy; TLE: *n* = 90; ETLE: *n* = 31 Total mean age onset = 10.03 ± 8.12; women = n.s.; men = n.s.	Total mean: n.s. Women: n.s. Men: n.s.	Mean ASMs = n.s.; women = n.s.; men = n.s. Surgery: no	WMS, LM subtest: delayed verbal memory recall WMS, VR subtest: delayed visual memory recall RAVLT: immediate verbal memory ROCF: delayed visuospatial memory 1 assessment: basal (T0)	*Women vs. men*: - N.s.d. delayed verbal memory (WMS) - >verbal learning, but n.s.d. in short-term memory or long-term memory evaluated by RAVLT - N.s.d. in delayed non-verbal memory (WMS) - < in delayed visuospatial memory (ROCF)
Strauss et al. [79]	Cross-sectional	*N* = 1.185 patients with epilepsy Women: *n* = 595 (50.21%) Men: *n* = 590 (49.79%)	Adults; total mean = 30.93 ± 9.65 years Women = n.s. Men = n.s.	Drug-resistant epilepsy; TLE: *n* = 1097; ETLE: *n* = 88 Total mean age onset = 11.91 ± 9.78 Women = n.s. Men = n.s.	Total mean: n.s. Women: n.s. Men: n.s.	Mean ASMs = n.s.; women = n.s.; men = n.s. Surgery: no	WMS-R: General memory, visual memory, verbal memory, and delayed memory 1 assessment: basal (T0)	*Women vs. men*: - N.s.d. in any memory variable
Trenerry et al. [80]	Longitudinal	*N* = 125 patients with epilepsy Women: *n* = 66 (52.80%) Men: *n* = 59 (47.20%)	Adults; total mean = 34.02 ± 9.38 years Women = 33.99 ± 8.71 years Men = 35.19 ± 8.81 years	Drug-resistant TLE with MTS Total mean age onset = 12.06 ± 9.44 years; women = 8.74 ± 7.94 years; men = 14.97 ± 11.12 years	Total mean: n.s. Women: n.s. Men: n.s.	Mean ASMs = n.s.; women = n.s.; men = n.s. Surgery: left and right temporal lobectomy	WMS-R, LM subtest: delayed verbal percentage recall 2 assessments: basal (T0) and after surgery (T1)	*Women vs. men*: - In left TLE: ↑ improvement in postoperative verbal memory. N.s.d. in preoperative verbal memory - In right TLE: n.s.d. in pre- and postoperative verbal memory
Trenerry et al. [81]	Longitudinal	*N* = 129 patients with epilepsy Women: *n* = 68 (52.71%) Men: *n* = 61 (47.29%)	Adults; total mean = 34.04 ± 9.51 Women = 33.69 ± 9.47 Men = 34.44 ± 9.54	Drug-resistant TLE Total mean age onset = 12.81 ± 9.66 years; women = 9.48 ± 8.02; men = 16.52 ± 11.48	Total mean: n.s. Women: n.s. Men: n.s.	Mean ASMs = n.s.; women = n.s.; men = n.s. Surgery: left and right temporal lobectomy	WMS-R, VR subtest: delayed visual percentage recall 2 assessments: basal (T0) and after surgery (T1)	*Women vs. men (total sample)*: - T0, T1 and change from T0 to T1: N.s.d. in delayed visual recall *Multiple regression*: - Women: size of the right hippocampus positively correlated with presurgical delayed visual percentage recall. The extirpation of a large right hippocampus was associated with delayed visual percentage decline - Men: size of the right hippocampus not correlated with delayed visual percentage recall - Women vs. men with right TLE: correlations of pre- and postsurgical delayed visual percentage recall change with the side of the right hippocampus (relatively to the left hippocampus) were significantly greater - Women vs. men with left TLE: T0: n.s.d. in the correlations between visual memory and the side of the right hippocampus (relatively to the left hippocampus)
Turon et al. [82]	Cross-sectional	*N* = 25 patients with epilepsy Women: *n* = 9 (36.00%) Men: *n* = 16 (64.00%)	Adults; total mean = 72.27 ± 7.19 years Women = n.s. Men = n.s.	TLE (64.00%), FLE (12.00%), multifocal (4.00%) and undetermined (20.00%) Total mean age onset = 71.92 ± 7.34 years; women = n.s.; men = n.s.	Total mean: n.s. Women: n.s. Men: n.s.	Mean ASMs = 2.2 ± 0.49; women = n.s.; men = n.s. Surgery: no	RAVLT: immediate and delayed verbal memory ROCF: immediate and delayed visual memory recall 1 assessment: basal (T0)	*Women vs. men*: - >verbal memory scores (composite domain index calculated using means from RAVLT) - N.s.d. in visual memory
Vascouto et al. [83]	Cross-sectional	*N* = 93 patients with epilepsy Women: *n* = 53 (57.00%) Men: *n* = 40 (43.00%)	Adults; total mean = 36.00 ± 1.00 years Women = n.s. Men = n.s.	Drug-resistant mesial TLE Total mean age onset = 9.00 ± 0.90; women = n.s.; men = n.s.	Total mean = 8.00 ± 0.80 Women: n.s. Men: n.s.	Mean ASMs = n.s; women = n.s.; men = n.s.; Monotherapy (37.60%); Polytherapy (62.4%) Surgery: no	RAVLT: verbal learning, immediate and delayed memory ROCF: copy and immediate visual memory recall WMS-III: LM and VPA I and II; VR I and II: immediate and delayed verbal memory; immediate and delayed visual memory WAIS-III, Digit Span: working memory 1 assessment: basal (T0)	*Women vs. men*: - N.s.d.
White et al. [84]	Longitudinal	*N* = 32 patients with epilepsy Women: *n* = 18 (56.25%) Men: *n* = 14 (43.75%)	Adults; total mean = 35 years; SD: n.s.; range: 18–57 years Women = n.s. Men = n.s.	Left TLE Total mean age onset = n.s.; women = n.s.; men = n.s.	Total mean: n.s. Women: n.s. Men: n.s.	Mean ASMs = n.s.; women = n.s.; men = n.s. Surgery: left ATL	WMS-R: LM and VPA I and II; VR I and II: immediate and delayed verbal memory; immediate and delayed visual memory RAVLT: verbal learning, immediate and delayed memory ROCF: immediate and delayed visual recall 2 assessments: basal (T0) and 6–10 months after surgery (T1)	*Women vs. men*: - From T0 to T1: ↓ postoperative decline (LM) and ↑ preservation in postoperative verbal memory (RAVLT) - N.s.d. in VPA and visual memory

AMIPB: Adult Memory & Information Processing Battery; ASM: antiseizure medication; ATL: anterior temporal lobectomy; AVLT: The Auditory Verbal Learning Test; BG: Bender-Gestalt Test; BMIPB: Memory and Information Processing Battery; BVRT: The Benton Visual Retention Test; BVRT-R: The Benton Visual Retention Test Revised; CAVLT: Children’s Auditory Verbal Learning Test; CBT: Corsi Block-Tapping Test; CVLT: California Verbal Learning Test; DCS-R: Diagnosticum für Cerebralschädigung - Revision; ETLE: extra temporal lobe epilepsy; FIAS: Focal impaired awareness seizure; FLE: frontal lobe epilepsy; HS: hippocampal sclerosis; IQ: Intellectual Quotient; LES: lesionectomy; LM: Logical Memory; MTS: mesial temporal sclerosis; N.r.: not reported; N.s.: not specified; n.s.d.: no significant differences; N.s.r.: no significant relationship; NVLT: Nonverbaler Lerntest; OLE: occipital lobe epilepsy; PAL: Paired-associate learning; RAVLT: Rey Auditory Verbal Learning Test; RCI: Reliable Change Index; ROCF: Rey-Osterreith Complex Figure Test; SAH: selective amygdalohippocampectomy; SRT: Story Recall Test; SOPT: Self-ordered Pointing Task; SS: Short Story Test; TLE: temporal lobe epilepsy; TPT: Tactual performance test; VLMT: Verbaler Lernund Merkfähigkeitstest; VPA: Verbal Paired Associates; VR: Visual Reproduction; VRPER: Visual Reproduction Percentage Retention; WAIS: Wechsler Adult Intelligence Scale; WAIS-R: Wechsler Adult Intelligence Scale-Revised; WB: Wechsler-Bellevue Form I; WISC: The Wechsler Intelligence Scale for Children; WMS-III: Wechsler Memory Scale third edition

### Evaluation of the quality of the studies

Regarding the evaluation of the quality of the studies included in the systematic review (Table 2), the percentage of compliance with the MMAT criteria ranged from 20% to 100%, depending on the study. Out of the 32 studies, 25 (78.13%) met the criterion of providing participants representative of the target population, while six studies (18.75%) were rated as unclear in this regard, and one (3.13%) did not meet this criterion. In terms of appropriate measurements regarding both the outcome and the intervention (or exposure), 28 studies (87.50%) met the criterion, with four studies (12.50%) receiving an unclear rating. Regarding the completeness of outcome data, 24 studies (75.00%) met the criterion, five studies (15.63%) were classified as unclear, and three studies (9.38%) did not meet this criterion. Concerning the consideration of confounders in the design and analysis, 22 studies (68.75%) met the criterion, seven studies (21.88%) received an unclear rating, and another three studies (9.38%) did not meet this criterion. Finally, regarding whether the intervention (or exposure) was administered as intended, all studies met the criterion.

**Table 2:** Risk of bias of the reviewed studies

References	Q1	Q2	Q3	Q4	Q5	% of compliance
Baxendale et al. [54]	Yes	Yes	Yes	Yes	Yes	100
Baxendale et al. [55]	No	Yes	Yes	Yes	Yes	80
Baxendale [56]	Yes	Yes	Yes	No	Yes	80
Bengner et al. [57]	Yes	Unclear	Yes	Yes	Yes	80
Bengston et al. [58]	Yes	Yes	Yes	Yes	Yes	100
Berenbaum et al. [59]	Yes	Yes	Yes	Unclear	Yes	80
Berger et al. [60]	Yes	Yes	Yes	Yes	Yes	100
Berger et al. [61]	Yes	Yes	Yes	Yes	Yes	100
Bjørnæs et al. [62]	Yes	Unclear	Yes	Unclear	Yes	60
Cánovas et al. [63]	Yes	Unclear	Yes	Unclear	Yes	60
Chai et al. [64]	Yes	Yes	Unclear	Yes	Yes	80
Davies et al. [65]	Yes	Yes	Yes	Yes	Yes	100
Grammaldo et al. [66]	Yes	Yes	Yes	Yes	Yes	100
Helmstaedter et al. [67]	Unclear	Yes	Yes	Yes	Yes	80
Helmstaedter et al. [68]	Yes	Yes	Yes	Yes	Yes	100
Hernández et al. [69]	Unclear	Yes	Yes	Yes	Yes	80
Höller et al. [70]	Unclear	Unclear	No	Unclear	Yes	20
Hudry et al. [71]	Yes	Yes	Yes	Unclear	Yes	80
Làdavas et al. [72]	Yes	Yes	Yes	No	Yes	80
Matešić et al. [73]	Yes	Yes	Yes	No	Yes	80
Operto et al. [52]	Yes	Yes	Yes	Yes	Yes	100
Salvato et al. [74]	Yes	Yes	Unclear	Yes	Yes	80
Schwartz and Dennerll [75]	Unclear	Yes	Unclear	Unclear	Yes	40
Smith et al. [76]	Unclear	Yes	Yes	Yes	Yes	80
Strauss et al. [77]	Yes	Yes	No	Yes	Yes	80
Strauss et al. [78]	Yes	Yes	Yes	Yes	Yes	100
Strauss et al. [79]	Yes	Yes	No	Yes	Yes	80
Trenerry et al. [80]	Yes	Yes	Yes	Yes	Yes	100
Trenerry et al. [81]	Yes	Yes	Yes	Unclear	Yes	80
Turon et al. [82]	Yes	Yes	Unclear	Yes	Yes	80
Vascouto et al. [83]	Yes	Yes	Unclear	Yes	Yes	80
White et al. [84]	Unclear	Yes	Yes	Yes	Yes	80

Q1: Are the participants representative of the target population?; Q2: Are measurements appropriate regarding both the outcome and intervention (or exposure)?; Q3: Are there complete outcome data?; Q4: Are the confounders accounted for in the design and analysis?; and Q5: During the study period, is the intervention administered (or exposure occurred) as intended?

### Qualitative analysis of reviewed studies

#### Characteristics of the sample (age group, epilepsy type, and treatment)

Concerning the epilepsy type, 21 of the 32 studies (65.63%) included exclusively patients with TLE, whereas 10 studies (31.25%) included mixed types of epilepsy (i.e., TLE and other types, mainly frontal lobe epilepsy [FLE]), and one study (3.13%) did not specify the type of epilepsy. Among the 21 studies that included only patients with TLE, five (23.81%) focused exclusively on mesial TLE and three (14.29%) focused exclusively on left TLE. Among the 10 studies with mixed types of epilepsy, four (40.00%) included both focal and generalized epilepsies.

Data on the side of seizure focus was available in 27 of the 32 studies (84.38%). Most of these studies included samples of patients with left and right-hemisphere epilepsy, four studies (14.81%) included exclusively left-hemisphere epilepsy, and two studies (7.41%) reported cases with left, right, and bilateral seizure onset.

Hemispheric dominance for language was reported in 14 of the 32 studies (43.75%), typically through the Wada test or functional MRI. Of them, nine studies (64.29%) included samples in which all participants exhibited typical left-hemispheric language dominance, whereas five studies (35.71%) included both typical (left) and atypical (right or bilateral) language representation.

Regarding the age group, four of the 32 reviewed studies (12.50%) included samples of children, whereas 28 (87.50%) included adult samples. Concerning sex, of the 32 studies, only one (3.13%) did not differentially report the sex of the participants, whereas the remaining 31 studies (96.88%) provided a total sample of 4,658 participants, of which 2,366 were female (50.79%), while 2,292 were male (49.21%).

Educational level was reported in 15 of the 32 studies (46.88%). However, among these, two studies (13.33%) provided incomplete information to calculate the mean years of education, one reported education using categorical variables (e.g., primary, secondary), and another presented data as median and interquartile range. Based on the remaining 13 studies (86.67%), the mean years of formal education across samples was 10.42 years.

Finally, 11 of the 32 studies (34.38%) provided data on patients treated exclusively with ASMs, while 13 studies (40.63%) reported data on patients who had undergone surgery, and eight studies (25.00%) did not specify the type of treatment for the patients. Among the 13 studies with patients who underwent surgery, only four (30.77%) indicated that patients were taking ASMs in addition to surgery for seizure control.

#### Memory assessment

Table 3 shows a summary of the instruments used for the evaluation of each memory domain. Working memory was explored in seven of the 32 reviewed studies (21.88%), with a digit span test being the most frequently used task. Immediate and delayed verbal memory were examined in 20 of the 32 studies (62.50%), most of them employing list learning tasks. Immediate visual memory was assessed in 20 of the 32 reviewed studies (62.50%), and delayed visual memory was examined in 16 out of the 32 studies (50.00%), with complex figure tests being the most frequently used.

**Table 3:** Summary of the instruments used for the evaluation of each memory domain and frequency of use

Memory domain	Instruments	Frequency of use
Working memory	Digit span test from WMS-III, WB, WAIS, or WISC	85.71%
	SOPT	14.29%
	BVRT-R	14.29%
	Memory tasks adapted from the Luria test	14.29%
	CBT	14.29%
Immediate verbal memory	List learning tasks from RAVLT, CVLT, CAVLT, AMIPB, and memory tasks adapted from the Luria test	80.00%
	Logical memory tasks from WMS, SRT, and AMIPB	35.00%
	Global memory index from the list learning and logical memory tasks of the AMIPB	5.00%
	PAL	5.00%
Delayed verbal memory	List learning tasks from RAVLT, CVLT, CAVLT, and memory tasks adapted from the Luria test	60.00%
	Logical memory tasks from WMS, SRT, AMIPB, and SS	55.00%
	Global memory index from the list learning and logical memory tasks of the AMIPB	5.00%
Immediate visual memory	ROCF	30.00%
	WMS	20.0%
	Design learning task from the AMIPB	15.0%
	DCS-R	10.00%
	Face recognition tasks	10.00%
	NVLT	5.00%
	Visual memory tasks adapted from Jones-Gotman	5.00%
	Visual and spatial navigation tasks assessed through a virtual reality adaptation of the Holeboard paradigm	5.00%
	Stick Test	5.00%
	BVRT	5.00%
	BG	5.00%
Delayed visual memory	ROCF and the complex figure from the AMIBP	50.00%
	WMS	37.50%
	Face recognition tasks	6.25%
	NVLT	6.25%
	Visual memory tasks adapted from Jones-Gotman	6.25%
	Visual and spatial navigation tasks assessed through a virtual reality adaptation of the Holeboard paradigm	6.25%

AMIPB: Adult Memory & Information Processing Battery; BG: Bender-Gestalt Test; BVRT: The Benton Visual Retention Test; BVRT-R: The Benton Visual Retention Test Revised; CAVLT: Children’s Auditory Verbal Learning Test; CBT: Corsi Block-Tapping Test; CVLT: California Verbal Learning Test; DCS-R: Diagnosticum für Cerebralschädigung – Revised; NVLT: Nonverbaler Lerntest; PAL: Paired-associate learning; ROCF: Rey-Osterreith Complex Figure Test; SOPT: Self-Ordered Pointing Task; SRT: Story Recall Test; SS: Short Story Test; WAIS: Wechsler Adult Intelligence Scale; WB: Wechsler-Bellevue Form I; WISC: The Wechsler Intelligence Scale for Children; WMS: Wechsler Memory Scale; WMS-III: Wechsler Memory Scale - Third Edition

Only two studies provided information on the proportion of men and women showing impaired memory performance according to established cutoff criteria [54, 55]. Using a cutoff of 2 SDs below the normative mean, Baxendale et al. [55] reported that 46.15% of men and 40.48% of women showed impaired verbal memory, whereas 12.50% of men and 20.00% of women exhibited impaired visual memory. Baxendale et al. [54] graphically presented cognitive profiles by gender and affected hemisphere, showing that all groups exhibited significant clinical deficits in verbal learning and retention, with most mean scores falling at or below the 10th percentile of the normative population and women having higher percentiles than men. In contrast, visual learning scores for all groups clustered around the 30th percentile, with men showing higher percentiles than women.

### Meta-analyses

#### Working memory

**Fig. 2:**
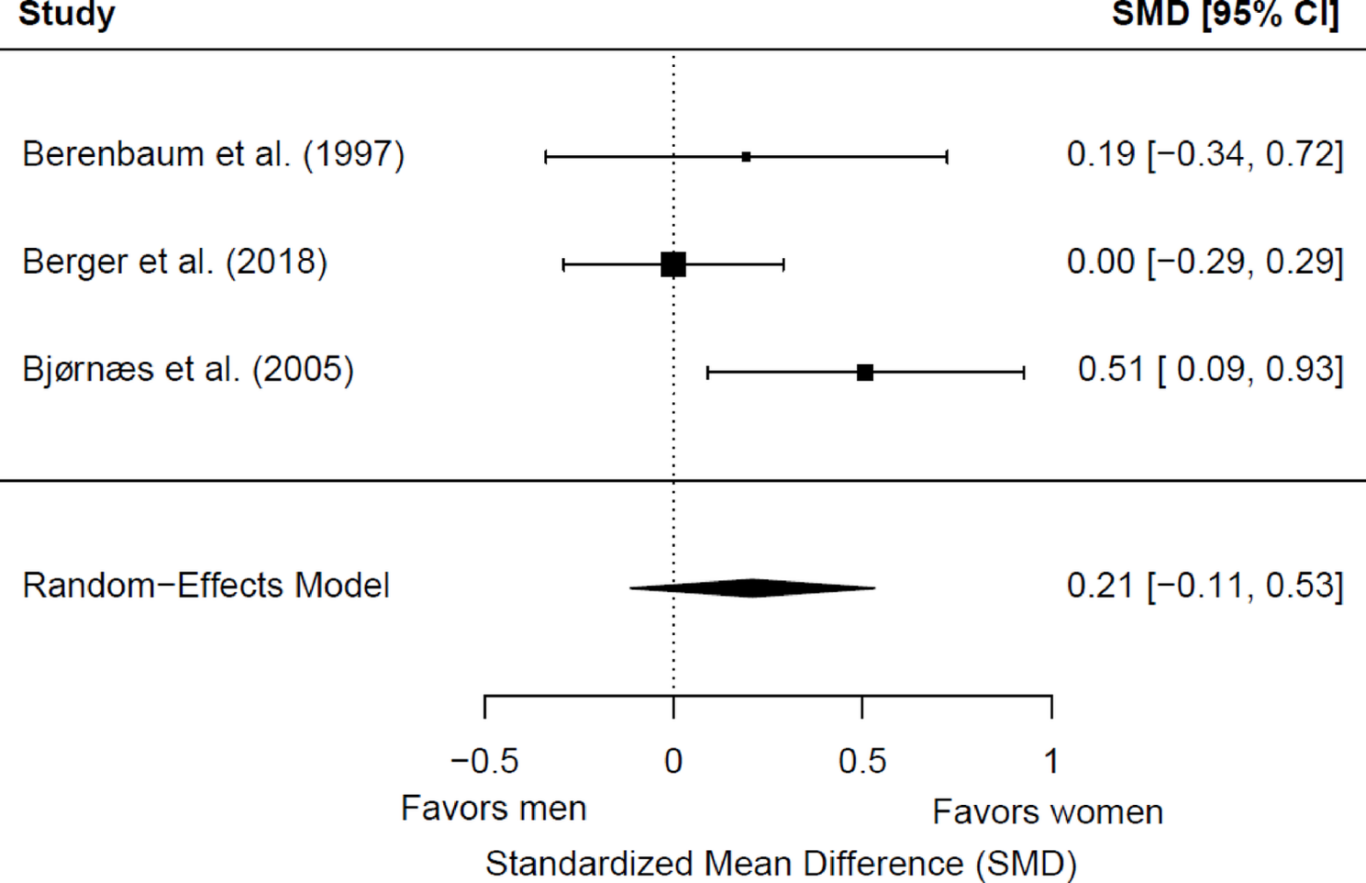
Forest plot for baseline differences in baseline working memory between women and men

We included three studies (329 patients with epilepsy; 165 women and 164 men) in the meta-analysis for baseline working memory. Of these, one (33.33%) showed a significant sex/gender difference. We found no significant differences between women and men (*g* = 0.21; 95% CI = −0.11, 0.53; *p* = 0.21) (Fig. 2). Heterogeneity was not significant (Q(2) = 3.85, *p* = 0.15; τ = 0.20; I² = 48.62%). No significant differences were found depending on epilepsy type (*p* = 0.51), the side of seizure focus (*p* = 0.77), or educational level (*p* = 0.55). All studies included adult patients with typical hemispheric dominance for language, so the potential moderator role of the age group and dominance could not be explored.

**Fig. 3:**
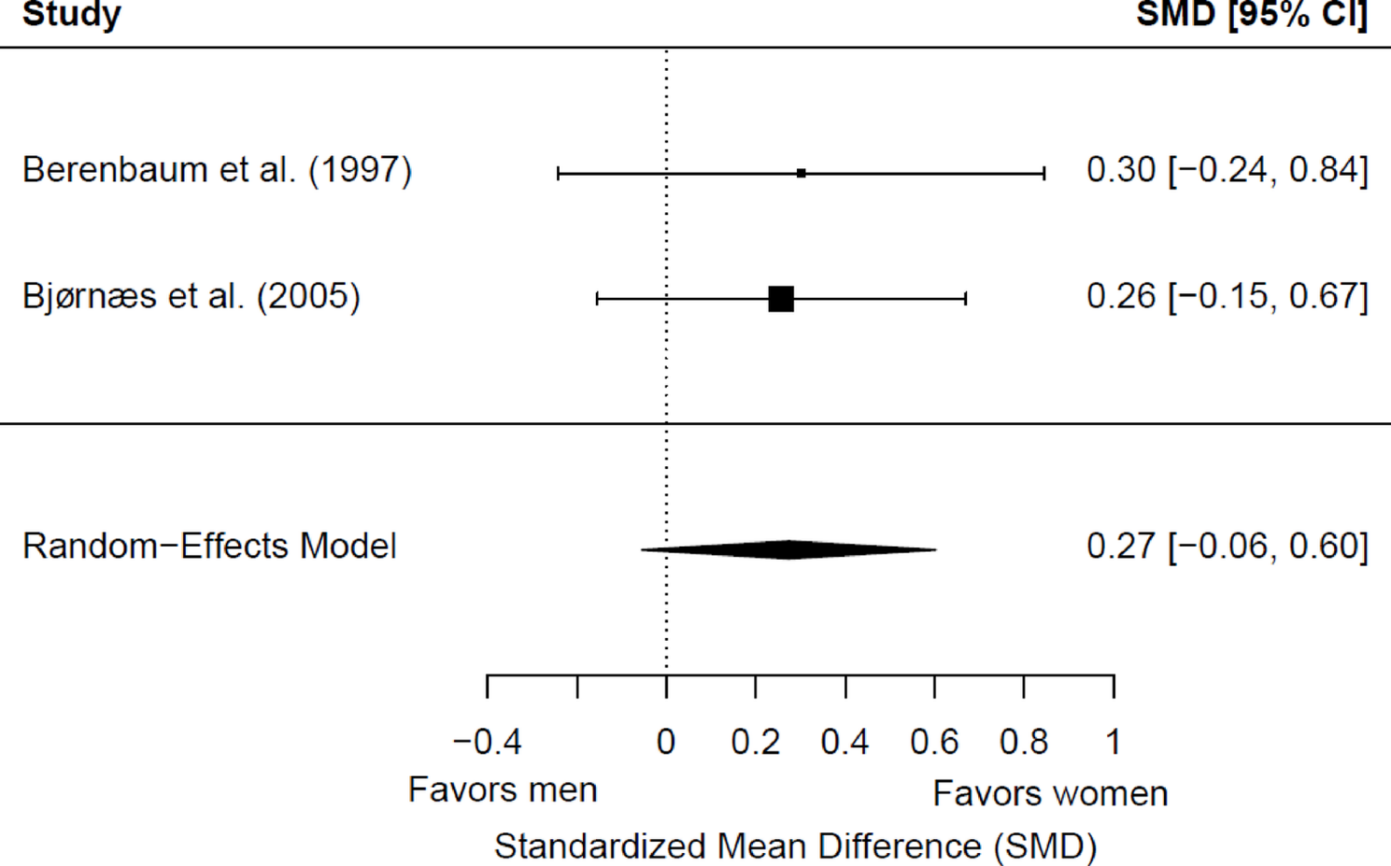
Forest plot for postsurgical differences in working memory between women and men

Two studies were included in the meta-analysis for postsurgical working memory (144 patients with epilepsy; 75 women and 69 men). Of these, no one showed a significant sex/gender difference. No significant differences were found between women and men (*g* = 0.27; 95% CI = −0.06, 0.60; *p* = 0.10) (Fig. 3). Heterogeneity was not significant (Q(1) = 0.02, *p* = 0.90; τ = 0.00; I² = 0.00%). No significant differences were found depending on the side of seizure focus (*p* = 0.77) or the educational level (*p* = 0.55). The two studies included adult patients exclusively with TLE with typical hemispheric dominance for language, so the potential moderator role of the epilepsy type, the dominance, and the age group could not be explored.

#### Immediate verbal memory

**Fig. 4:**
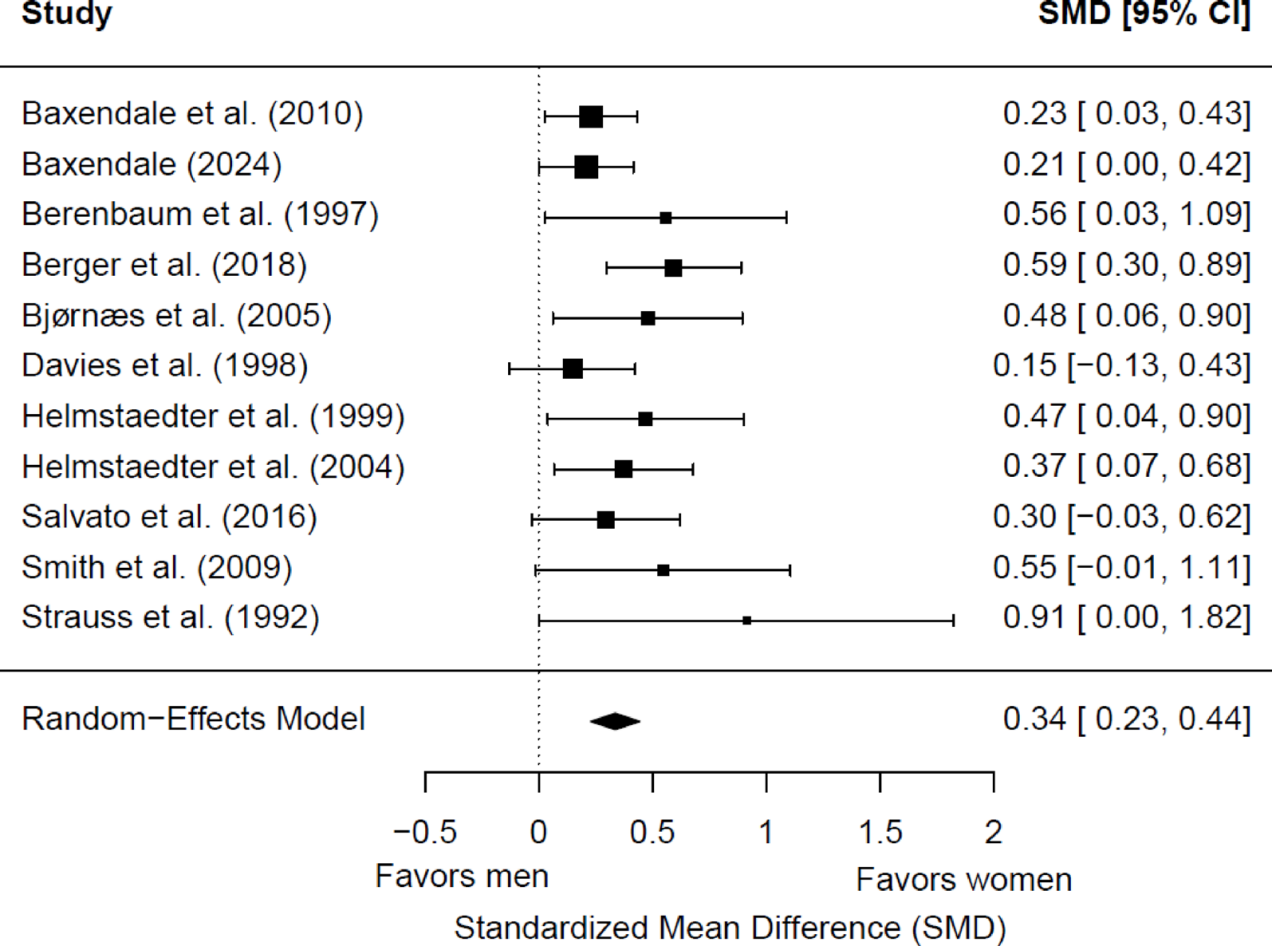
Forest plot for baseline differences in immediate verbal memory between women and men

We initially included 12 studies (1,935 patients with epilepsy; 1,003 women and 932 men) in the meta-analysis for baseline immediate verbal memory. Of these, six (50%) showed a significant sex/gender difference, and two (16.7%) showed a marginally significant effect. Women had significantly better memory scores than men (*g* = 0.31; 95% CI = 0.19, 0.43; *p* < 0.0001). Heterogeneity was not significant (Q(11) = 17.14, *p* = 0.10; τ = 0.13; I² = 37.40%). Egger’s test suggested potential publication bias (b = 0.02; 95% CI = −0.24, 0.28; z = 2.49, *p* = 0.01). A trim-and-fill procedure estimated four potentially missing studies, yielding an adjusted but still significant effect (*g* = 0.24; 95% CI = 0.11, 0.37; *p* = 0.0002), although heterogeneity increased (Q(15) = 29.50, *p* = 0.01; τ² = 0.17; I² = 48.85%). To assess the robustness of this finding, we conducted a sensitivity analysis excluding one outlier study [60], identified using a Galbraith plot. The resulting model, based on 11 studies (1,758 patients; 908 women and 850 men), showed a slightly stronger effect size (*g* = 0.34; 95% CI = 0.23, 0.44; *p* < 0.0001) (Fig. 4) and substantially reduced heterogeneity (Q(10) = 10.84, *p* = 0.37; τ² = 0.07; I² = 15.18%). The effect size was significantly moderated by epilepsy type (*p* = 0.017): in studies including only patients with TLE, the effect size favoring women (*g* = 0.28; 95% CI 0.17, 0.38) was significantly smaller than in studies with mixed or other epilepsy types (g = 0.61; 95% CI 0.36, 0.86). No significant differences were found based on the side of seizure focus (*p* = 0.27), the hemispheric dominance for language (*p* = 0.32), the age group (*p* = 0.46), or the educational level (*p* = 0.25). It is worth noting that four of the eleven studies (36.36%) did not report data on language dominance [54, 56, 61, 76], and six studies (54.55%) did not provide information on educational level [54, 56, 67, 68, 76, 77].

**Fig. 5:**
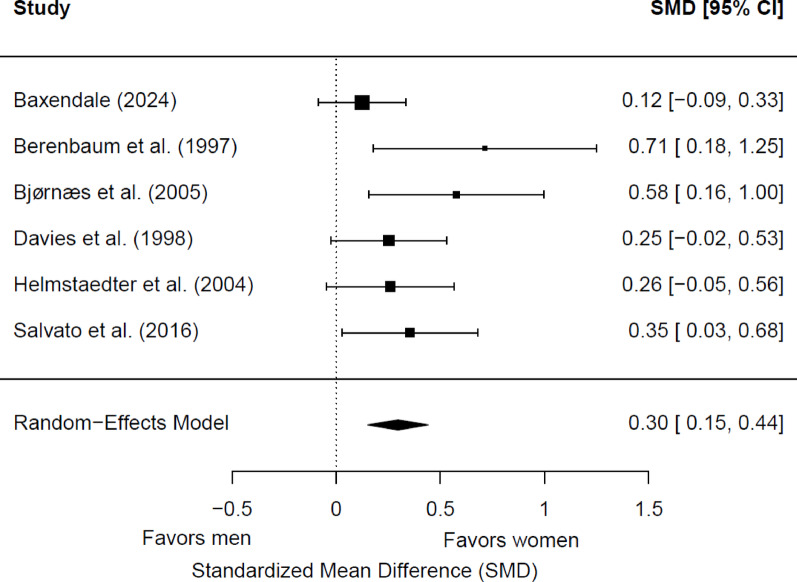
Forest plot for postsurgical differences in immediate verbal memory between women and men

Six studies (1,025 patients; 518 women and 507 men) were included in the meta-analysis for postsurgical immediate verbal memory. Of these, three (50%) showed a significant sex/gender difference. Women had significantly better memory scores than men (*g* = 0.30; 95% CI = 0.15, 0.44; *p* < 0.0001) (Fig. 5). Heterogeneity was not significant (Q(5) = 6.82, *p* = 0.23; τ = 0.09; I² = 22.01%). No significant differences were found based on the side of seizure focus (*p* = 0.68), the hemispheric dominance for language (*p* = 0.46), and the educational level (*p* = 0.10). One study did not report data on language dominance [56], and two did not provide information on educational level [56, 68]. All studies included adult patients with TLE, so the potential moderator role of epilepsy type or age group could not be examined.

#### Delayed verbal memory

**Fig. 6:**
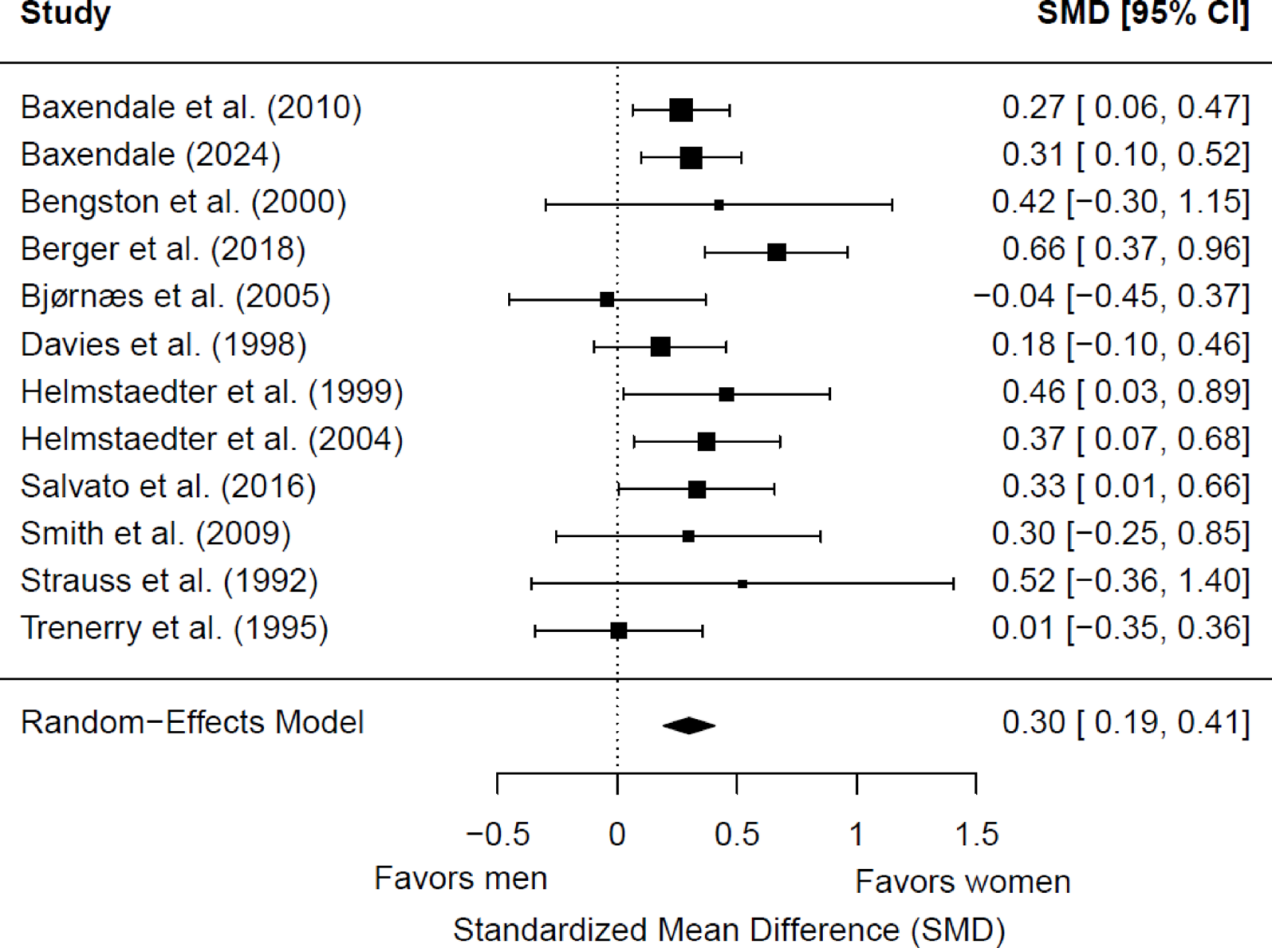
Forest plot for baseline differences in delayed verbal memory between women and men

We included 12 studies (1,850 patients with epilepsy; 957 women and 893 men) in the meta-analysis for baseline delayed verbal memory. Of these, six (50%) showed a significant sex/gender difference. Women had significantly better memory scores than men (*g* = 0.30; 95% CI = 0.19, 0.41; *p* < 0.0001) (Fig. 6). Heterogeneity was not significant (Q(11) = 13.13, *p* = 0.29; τ = 0.08; I² = 19.35%). Egger’s test suggested no potential publication bias (b = 0.27; 95% CI = −0.03, 0.57; z = 0.22, *p* = 0.83). The effect size was significantly moderated by epilepsy type (*p* = 0.02). In studies that included only patients with TLE, the effect size favoring women (*g* = 0.26; 95% CI = 0.16, 0.36) was significantly smaller than in studies with mixed or other epilepsy types (*g* = 0.58; 95% CI = 0.33, 0.83). No significant differences were found depending on the side of seizure focus (*p* = 0.31), the hemispheric dominance for language (*p* = 0.09), the age group (*p* = 0.99), or the educational level (*p* = 0.45). Four studies (33.33%) did not report data on language dominance [54, 56, 61, 76], and six studies (50.00%) did not provide information on educational level [54, 56, 67, 68, 76, 77].

**Fig. 7:**
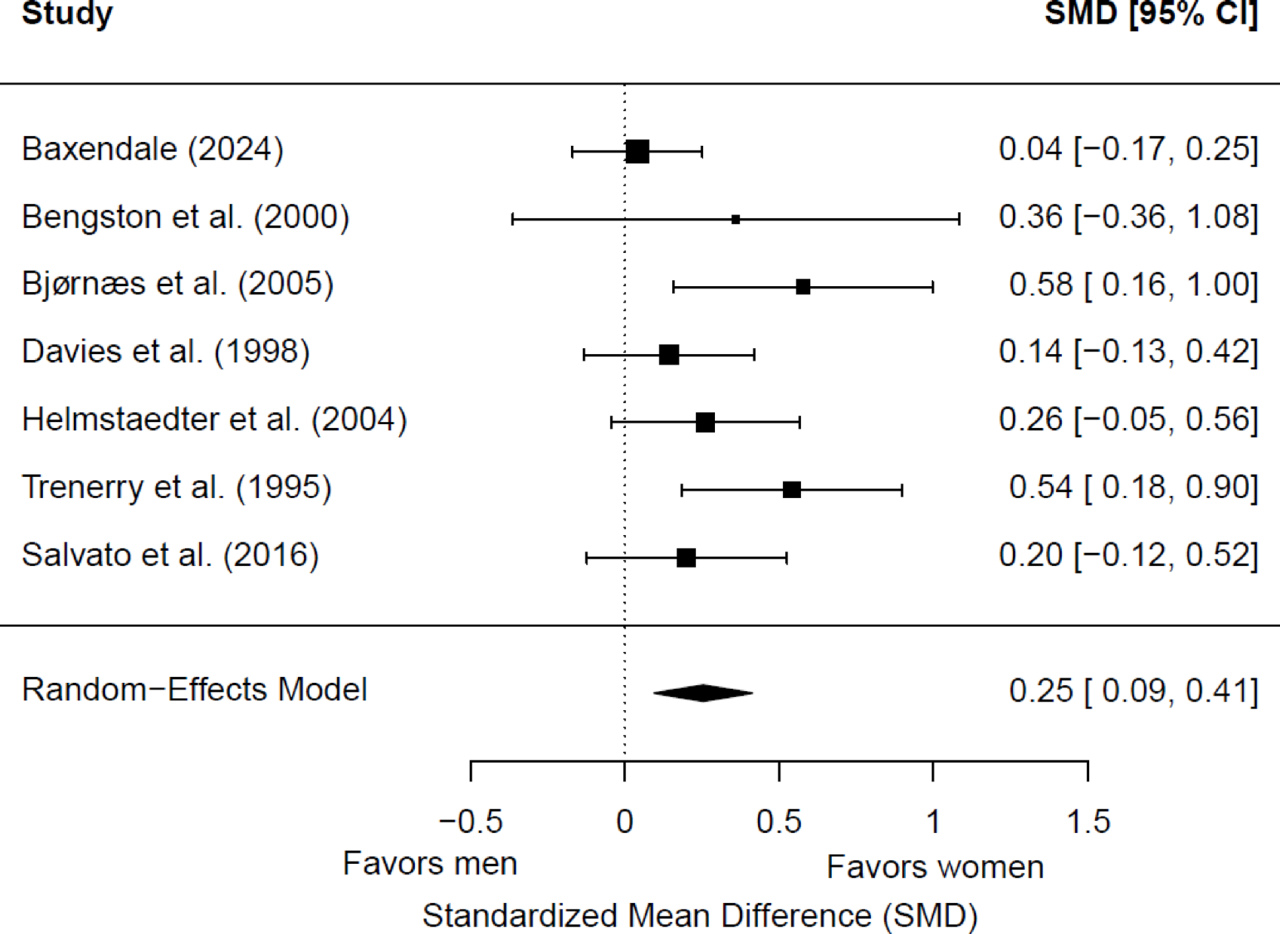
Forest plot for postsurgical differences in delayed verbal memory between women and men

Seven studies (1,120 patients; 567 women and 553 men) were included in the meta-analysis for postsurgical delayed memory. Of these, two (28.57%) showed a significant sex/gender difference. Women had significantly better memory scores than men (*g* = 0.25; 95% CI = 0.09, 0.41; *p* = 0.0018) (Fig. 7). Heterogeneity was not significant (Q(6) = 9.23, *p* = 0.16; τ = 0.13; I² = 38.50%). The effect size was significantly moderated by the side of seizure focus: studies including a greater proportion of patients with left-hemisphere epilepsy showed poorer postsurgical delayed verbal memory (*p* = 0.047). No significant differences were found depending on the hemispheric dominance for language (*p* = 0.74) or the educational level (*p* = 0.14). One study did not report data on language dominance [56], and two did not provide information on educational level [56, 68]. All studies included adult patients with TLE, so the potential moderator role of the epilepsy type or age group could not be analyzed.

#### Immediate visual memory

**Fig. 8:**
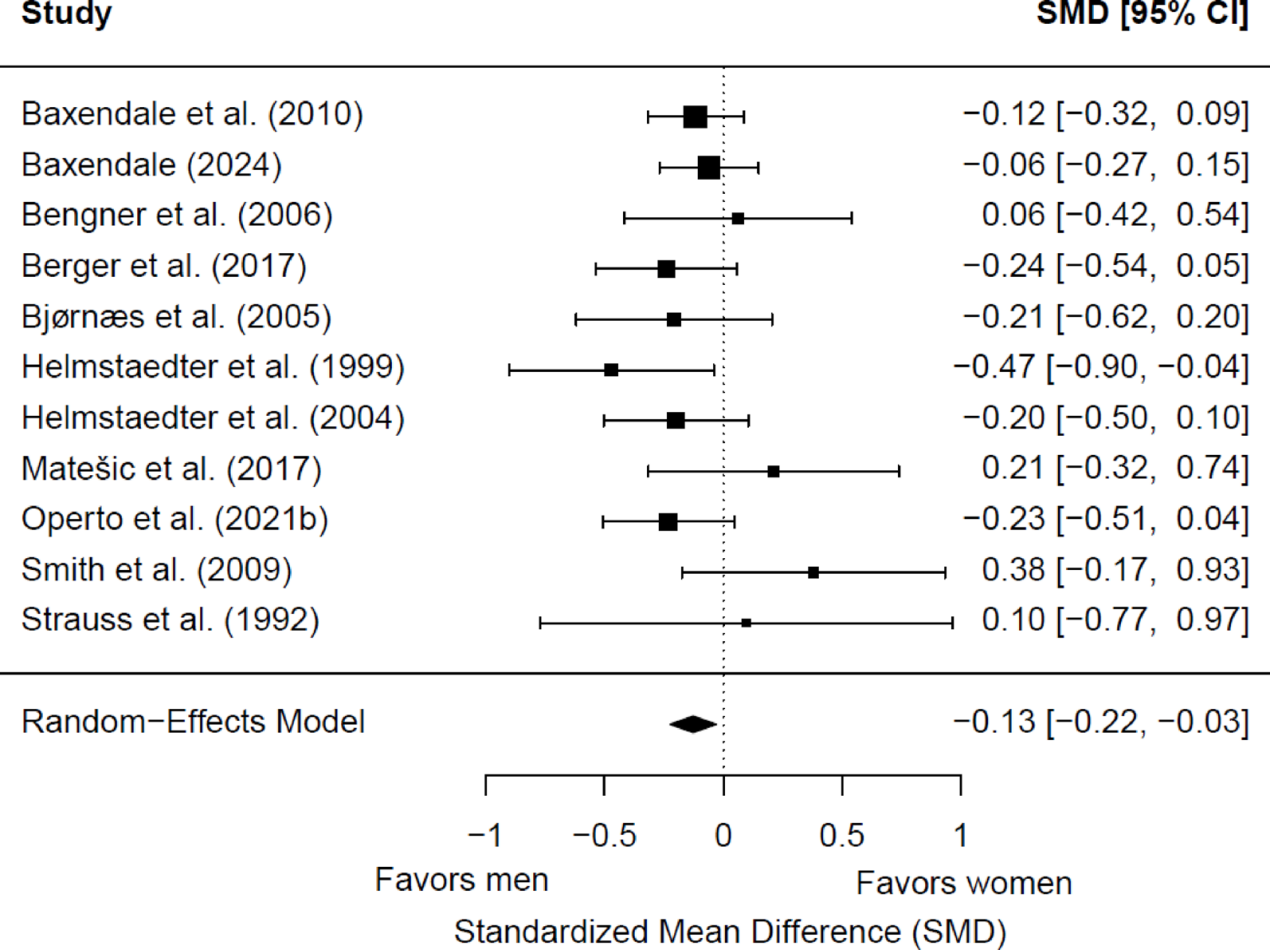
Forest plot for baseline differences in immediate visual memory between women and men

Eleven studies (1,663 patients with epilepsy; 861 women and 802 men) were included in the meta-analysis for baseline immediate visual memory. Of these, one (9.09%) showed a significant sex difference. Men had significantly better memory scores than women (*g* = −0.13; 95% CI = −0.22, −0.03; *p* = 0.01) (Fig. 8). Heterogeneity was not significant (Q(10) = 9.99, *p* = 0.44; τ = 0.01; I² = 0.02%). Egger’s test suggested no potential publication bias (b = −0.24; 95% CI = −0.50, 0.02; z = 0.91; *p* = 0.36). No significant differences were found depending on the epilepsy type (*p* = 0.51), the side of seizure focus (*p* = 0.20), the hemispheric dominance for language (*p* = 0.92), and the age group (*p* = 0.88). Only two studies reported data on educational level [60, 62], so no meta-regression analyses were carried out depending on this variable.

**Fig. 9:**
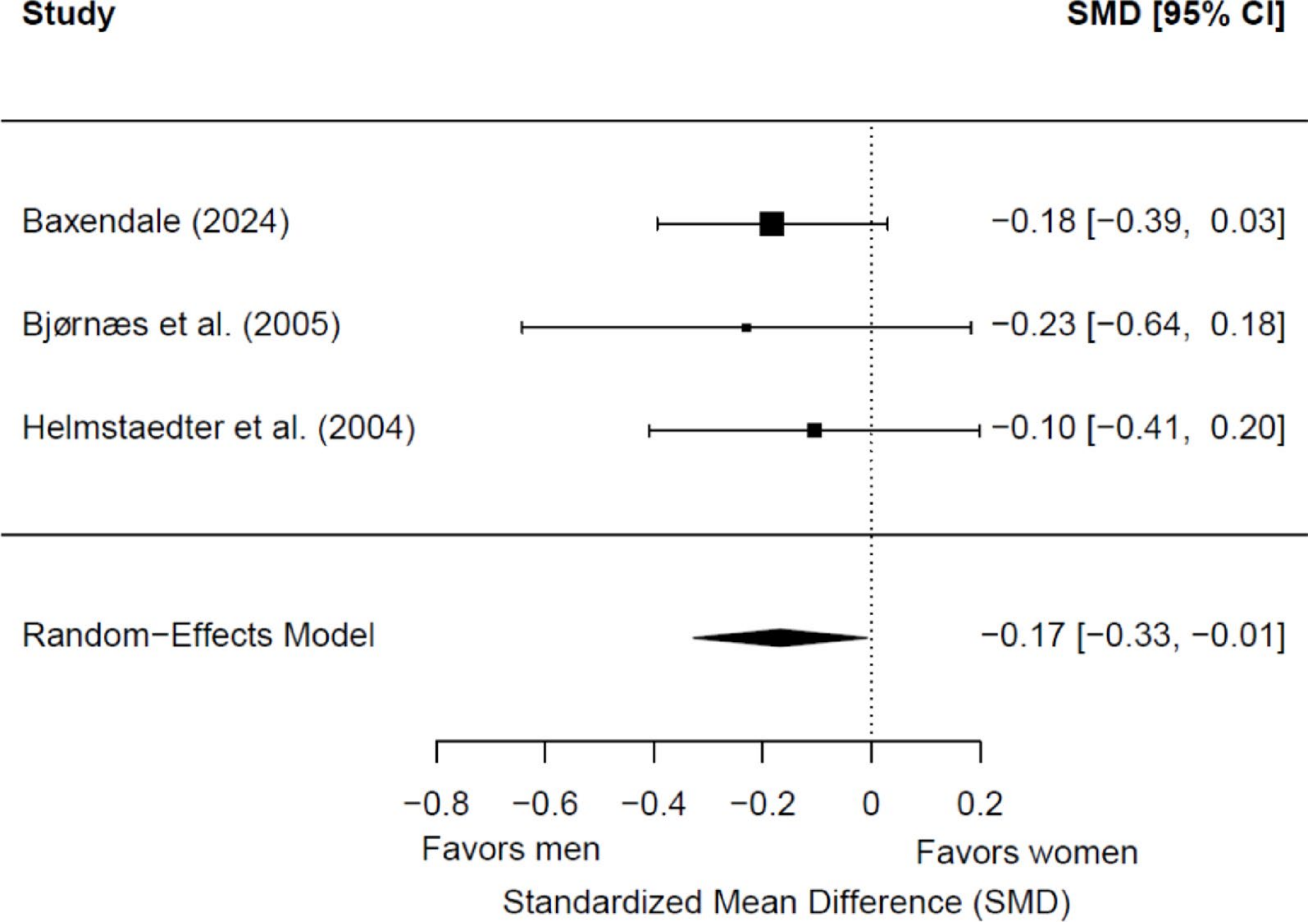
Forest plot for postsurgical differences in immediate visual memory between women and men

Three studies (608 patients; 309 women and 299 men) were included in the meta-analysis for postsurgical immediate visual memory. Of these, no one showed a significant sex/gender difference. Men had significantly better memory scores than women (*g* = −0.17; 95% CI = −0.33, −0.01; *p* = 0.04) (Fig. 9). Heterogeneity was not significant (Q(2) = 0.27, *p* = 0.87; τ = 0.01; I² = 0.00%). No significant differences were found depending on the side of seizure focus (*p* = 0.61). All studies included adult patients with TLE, so no subgroup analyses by epilepsy type or age group were carried out. Only two studies reported data on hemispheric dominance for language [62, 68], and only one reported data on educational level [62], so no meta-regression analyses could be carried out depending on these variables.

#### Delayed visual memory

**Fig. 10:**
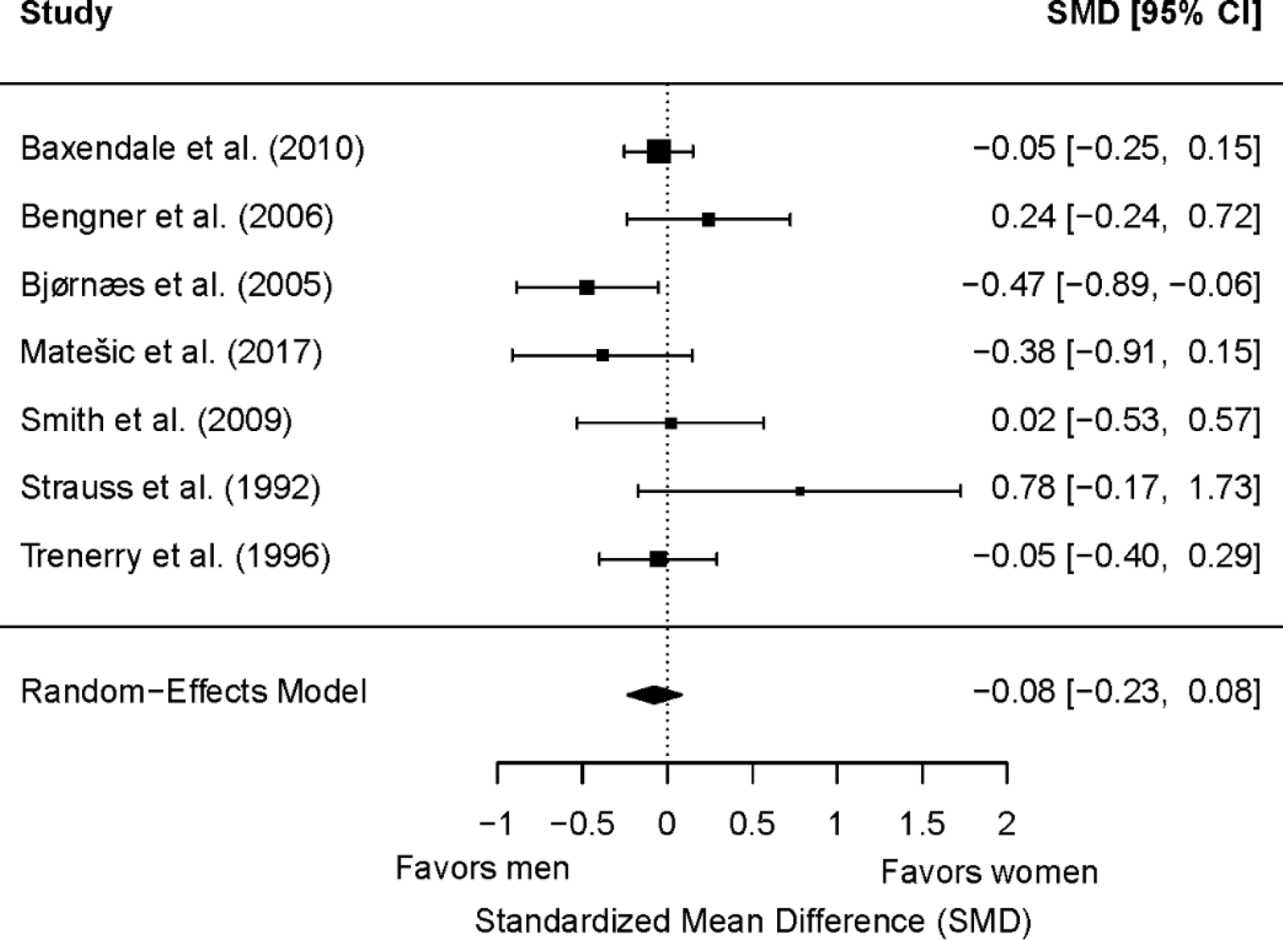
Forest plot for baseline differences in delayed visual memory between women and men

Seven studies (799 patients with epilepsy; 438 women and 361 men) were included in the meta-analysis for baseline delayed visual memory. Of these, one (14.29%) showed a significant sex/gender difference. No significant differences were found between men and women in memory scores (*g* = −0.08; 95% CI = −0.23, 0.08; *p* = 0.34) (Fig. 10). Heterogeneity was not significant (Q(6) = 9.76, *p* = 0.14; τ = 0.07; I² = 10.88%). The effect size tended to be moderated by epilepsy type (*p* = 0.06). Although differences were not statistically significant for either TLE or mixed/other epilepsy types, the effect size favored men in patients with TLE (*g* = −0.16; 95% CI = −0.35, 0.03) and favored women in patients with mixed or other epilepsy types (*g* = 0.23; 95% CI = −0.13, 0.59). No significant differences were found depending on the side of seizure focus (*p* = 0.23) or the age group (*p* = 0.76). Only three studies reported data on hemispheric dominance for language [62, 77, 81], with the total sample having typical language dominance in two of these studies, and only two reported data on educational level [62, 81], so no meta-regression analyses could be carried out depending on these variables.

**Fig. 11:**
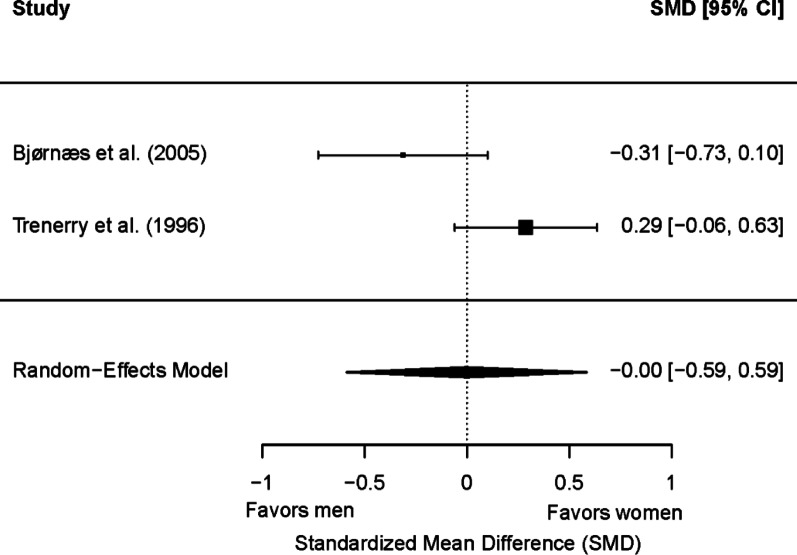
Forest plot for postsurgical differences in delayed visual memory between women and men

Two studies (220 patients; 114 women and 106 men) were included in the meta-analysis for postsurgical delayed memory. Of these, no one showed a significant sex/gender difference. No significant differences were found between men and women in memory scores (*g* = −0.00; 95% CI = −0.59, 0.59; *p* = 0.99) (Figure 11). Heterogeneity was significant (Q(1) = 4.73, *p* = 0.03; τ = 0.38; I² = 78.88%), but no outlier studies were identified using the Galbraith plot. The two studies included adult patients with TLE and typical hemispheric dominance for language. Due to the limited number of studies in this meta-analysis, the potential moderator effects of epilepsy type, side of seizure focus, and educational level could not be examined.

## Discussion

This meta-analysis synthesized data from 32 studies with patients with epilepsy to examine the potential existence of sex-gender differences across various memory domains. Results showed that women had an advantage in both immediate and delayed verbal memory that was maintained even after epilepsy surgery. In contrast, men exhibited superior performance in immediate visual memory, a pattern observed throughout pre- and postsurgical assessments. However, no sex-gender differences were found in delayed visual memory and working memory.

Regarding working memory, no significant sex-gender differences were found at either baseline or postsurgical assessments. Importantly, only three studies assessed this domain at baseline, and just two studies provided postsurgical data, all of them in adult patients with typical dominance for language. Moreover, only one study incorporated mixed epilepsy types [61] and one study included exclusively patients with left-sided seizure focus [59]. Although all studies included in the meta-analysis employed the same task to assess working memory (i.e., digit span), the small number of available studies limits statistical power to reliably detect sex-gender differences and to explore potential moderators with sufficient reliability. From a neuroanatomical perspective, working memory primarily involves the activation of dorsolateral prefrontal, frontoparietal, and additional frontal regions [85], highlighting the importance of examining how disruptions across different neural networks (e.g., epilepsy types) may interact with sex-gender to affect working memory outcomes. Moreover, future research should address these questions in pediatric populations, which remain understudied to date.

Meta-analyses showed a presurgical advantage for women in immediate and delayed verbal memory, with moderate effect sizes. This suggests that women had a higher resilience to the impact of epilepsy on verbal memory, not only for encoding information but also for long-term consolidation. The observed differences may be attributed to multiple neurocognitive, functional, and hormonal factors. Specifically, it has been proposed that women more frequently employ semantic clustering strategies during verbal memory tasks, which enhance encoding efficiency even in the presence of hippocampal dysfunction [54, 59]. This more organized and effective encoding style has been associated with increased activation in frontal regions and reduced reliance on mesial temporal structures, potentially contributing to the women’s advantage in verbal memory [61]. In addition, hormonal influences may explain these differences [68, 77]. Specifically, estrogens may enhance the recruitment of frontal regions and improve the efficiency of neural networks involved in verbal processing [61]. Interestingly, we found that epilepsy type significantly moderated the effect size in both immediate and delayed verbal memory before surgery. Although sex-gender differences in verbal memory were significant in both studies with patients with TLE and studies with mixed samples, the effect size was significantly larger in studies with mixed samples. This pattern may reflect the broader variability in neural network disruptions across different epilepsy syndromes. In more heterogeneous samples, where memory performance may be impacted by dysfunction in multiple interconnected brain systems beyond the hippocampus, the protective role of women’s more efficient encoding strategies and greater recruitment of frontal resources may become even more prominent, thereby amplifying sex-gender differences in verbal memory performance.

Meta-regression analyses showed that sex-gender differences in verbal memory did not vary depending on the age group or the educational level. It should be noted that only one study with pediatric population was included in the meta-analysis of baseline memory differences [76], which substantially limits the possibility of detecting potential age-related effects. From a developmental perspective, it has been proposed that the female brain matures earlier than the male brain, potentially providing a cognitive advantage and better preservation of verbal functions following early injury [77]. Age could theoretically moderate sex-gender differences in verbal memory in epilepsy, given that brain plasticity and the capacity for functional reorganization vary across development [86, 87], but more studies are needed to clarify this issue. Regarding educational level, it is important to note that not all studies reported this information. Among those that did, no sex or gender differences were observed. The absence of a moderating effect may therefore reflect the relative homogeneity of this variable between men and women across samples, with limited variability in years of education (approximately ranging from 9.3 to 12.9 years).

Our meta-analyses showed that the women’s advantage for verbal memory persisted after surgery. Accordingly, it has been suggested women’s brains have a greater capacity for interhemispheric reorganization of memory functions compared to men, which may help to preserve verbal memory by redistributing functions to other cortical areas after surgery [56, 60, 68]. It should be noted that these postsurgical analyses were based exclusively on studies of adult patients with TLE, which precludes a more detailed exploration of potential moderating variables, such as epilepsy type or age. The effect size for postsurgical delayed verbal memory was moderated by seizure focus: studies with a higher proportion of left-hemisphere epilepsy cases showed poorer outcomes. This moderating effect was specific to postsurgical delayed verbal memory, which is highly dependent on left temporal lobe integrity [88, 89]. For other verbal memory outcomes, this variable did not reach significance, likely reflecting the relative homogeneity across studies in the proportion of men and women with left- and right-hemisphere epilepsy. Similarly, hemispheric dominance for language did not significantly moderate the results, although this finding should be interpreted cautiously given the limited variability and incomplete reporting across studies.

Regarding visual memory, an advantage for men was observed in immediate visual memory during the presurgical assessment, which persisted after surgery, with small effect sizes in both cases. Interestingly, this advantage was not influenced by epilepsy type, side of surgery, hemispheric dominance for language or age before surgery, suggesting a potentially generalizable pattern across different clinical contexts. The potential moderator effect of educational level could not be explored due to the limited studies reporting data on this variable. After surgery, studies examining visual memory included only adult patients with TLE. These results are consistent with those found in the general population, which attribute greater efficiency in visual and visuospatial tasks to men [90, 91]. This advantage for men has been interpreted, at least in part, as a result of a higher functional lateralization in men, who may preserve the visuospatial specialization of the right hemisphere when language reorganization occurs following left-hemispheric damage [62]. In contrast, women with atypical language dominance often exhibit more bilateral language representation after left-hemispheric insult, which may support verbal memory performance by recruiting right-hemispheric resources, but at the cost of a “crowding” effect where verbal and visuospatial functions compete for shared neural substrates, potentially compromising visual memory [62, 67, 68, 77]. According to our results, findings suggest that sex and gender differences are more robust in verbal memory than in visual memory [90].

Our meta-analysis showed no significant sex-gender differences in delayed visual memory at either neuropsychological assessment timepoint. However, a moderating trend by epilepsy type was noted in studies for presurgical delayed visual memory: the advantage tended to favor men in patients with TLE and to women in those with mixed or other epilepsy types. One possible explanation is that in patients with TLE, particularly with left-hemispheric foci, language reorganization often involves right-hemispheric structures, potentially interfering with visuospatial processing in women and reducing their performance on visual memory tasks [57, 67, 68]. In contrast, in more heterogeneous samples that include patients with non-temporal or extratemporal epilepsies, language reorganization may be less frequent or involve different neural networks [92], allowing women to maintain a relative advantage in visual memory. The results found in the present meta-analysis could be influenced by the heterogeneity of the tasks (i.e., figural or face tasks) used or even the response modality (recognition versus free recall) [60, 76]. Furthermore, some visual memory tests may fail to adequately differentiate between purely visual and verbally mediated memory [60, 73], which could mask potential sex-gender differences. Moreover, our meta-analysis did not identify sex-gender differences in baseline delayed visual memory as a function of side of seizure focus and age group. However, this last finding should be interpreted with caution, as only one study included a pediatric sample [76]. Language dominance and education could not be tested as moderators for delayed visual memory because of limited data.

This study has several strengths, including the comprehensive analysis of multiple memory domains at both baseline and postsurgical timepoints, the consideration of adult and pediatric samples, and the inclusion of terms related to sex hormones in the search strategy, alongside “sex” and “gender”, to capture the potential neurobiological basis of the sex-gender differences in this population. Importantly, this is the first meta-analysis examining sex-gender differences in memory among patients with epilepsy. Nonetheless, several important limitations must be acknowledged. First, the available evidence was uneven across memory domains, and results from meta-analyses based on fewer than five outcomes should be interpreted with caution due to limited statistical power [93]. Second, most of the included samples consisted of adults with TLE, which restricts the generalizability of the findings to other epilepsy types and pediatric populations. In addition, despite most meta-analyses showed no significant heterogeneity, assessment tools were variable across studies. Third, the lack of systematic reporting of clinical variables such as seizure frequency, the presence of hippocampal sclerosis, psychiatric comorbidities, and pharmacological treatment, as well as demographic variables such as educational level limits the possibility of analyzing their interaction with sex-gender differences. Fourth, none of the studies explicitly addressed the influence of hormonal cycles, despite their potential impact on cognitive performance and cerebral activation. Finally, only two studies provided data on the proportion of men and women meeting established cutoff criteria for memory impairment, restricting the possibility of assessing the clinical implications of sex/gender differences for neuropsychological practice.

Despite these limitations, our findings show a moderate advantage for women with epilepsy in immediate and delayed verbal memory tasks, which persists after surgery, as well as a small advantage for men with epilepsy in immediate visual memory tasks at baseline and after surgery. No significant sex-gender differences were detected in working memory or delayed visual memory. Considering the findings of the reviewed studies, it is possible to make certain recommendations for future studies. First, the results support the relevance of systematically including sex and gender as moderating variables in the neuropsychological assessment of people with epilepsy. Taking sex and gender differences into account could improve the accuracy of neuropsychological assessments and presurgical predictions, as well as contribute to the development of more personalized strategies in cognitive and surgical intervention. Second, clinical variables such as seizure frequency, epilepsy type, side of seizure focus, ASMs, comorbidities, and hormonal cycle should be consistently recorded to elucidate the underlying mechanisms of the potential sex-gender differences. Third, future studies should improve the specificity of neuropsychological measures used to assess visual memory, avoiding verbally mediated memory tasks. Fourth, pediatric populations remain largely underrepresented in this field. Given developmental differences in brain plasticity and reorganization, longitudinal studies including children and adolescents are essential to better understand how sex-gender differences emerge and evolve across the lifespan. In fact, in both adult and pediatric samples, 53.1% of studies were cross-sectional and 46.9% longitudinal, with most of the longitudinal studies focusing on surgery effects within a relatively short timeframe - typically between six months and one year after surgery. This highlights a gap in our understanding of the long-term evolution of sex-gender differences in memory performance. Future research should prioritize longitudinal studies with extended follow-up periods that capture the trajectory of cognitive outcomes over time and across different epilepsy populations, beyond the surgical context.

## Data Availability

This review is registered in PROSPERO (CRD420251006928). The datasets used and/or analyzed during the current study are available from the corresponding author on reasonable request.
